# Numeric Analysis of Polarized Lidar Contrast Enhancement Across Diverse Targets in Fog

**DOI:** 10.3390/s26165053

**Published:** 2026-08-09

**Authors:** Manuel Petzi, Dominik Reitzle, Alwin Kienle

**Affiliations:** 1 Institut für Lasertechnologien in der Medizin und Meßtechnik an der Universität Ulm, Helmholtzstr. 12, D-89081 Ulm, Germany; dominik.reitzle@ilm-ulm.de (D.R.); alwin.kienle@ilm-ulm.de (A.K.); 2 Faculty of Natural Sciences, Universität Ulm, D-89081 Ulm, Germany

**Keywords:** lidar, polarization, turbid media, light scattering, radiative transfer, Monte Carlo simulation

## Abstract

Lidar systems are of major importance for driver assistance systems and autonomous driving, but their obstacle detection range can be heavily impaired by adverse weather conditions like fog. To mitigate the effects of fog, the use of polarized light has been proposed. We simulated a polarized lidar system, taking Mie theory-based scattering functions for different fog types at several wavelengths into account, and investigated the possible increase in contrast between fog backscattering and target returns for different surface types, depending on the target orientation and the polarizer configuration. Fog is modeled as an infinite, homogeneous, scattering, and absorbing medium. The simulated detector registers the time-dependent radiance, resolved by scattering order. Our findings show that the suitable choice of illumination and detection polarization allows the amount of single-scattered light detected to be reduced by two to four orders of magnitude and the contrast between fog and target to be increased significantly. Crucially, this approach delivers robust contrast enhancement regardless of whether the target surface is depolarizing Lambertian or perfectly reflecting.

## 1. Introduction

Lidar is a versatile and widespread method to determine distances by means of light propagation. Applications range from atmospheric science [1,2,3,4,5], over geodesy [6,7,8], to distance measurement and obstacle detection for vehicles and robots [9,10,11,12,13,14,15].

Recently, the importance of lidar systems in the automotive context has increased due to their use in driver assistance systems and autonomous driving. However, under adverse weather conditions like fog, rain, snow, or dust, the performance of lidar systems and cameras is degraded due to the light scattering in these media. The obstacle detection range can be significantly reduced, which impacts road safety. A lower speed is necessary to allow timely braking. Defogging techniques can be used to mitigate the effects of adverse weather on cameras [16,17]. For lidar systems, taking the light’s polarization into account has been suggested as a way to filter out most of the single-scattered light, and thereby increase the contrast between the fog and obstacles [18,19,20,21]. Circularly polarized (CP) light seems to be advantageous as it depolarizes slower under the influence of multiple scattering events than linearly polarized light [22,23] does.

The suitable framework to describe light propagation in turbid media on a mesoscopic length scale is radiative transfer theory [24,25]. It is applicable if interference effects can be neglected and the scattering events are independent [26]. The light’s polarization is accounted for in the vectorial radiative transfer Equation (vRTE) by means of the Stokes formalism. A very flexible way to obtain numeric solutions to the vRTE is Monte Carlo simulations [27,28,29].

Our goal in this work is to simulate a lidar system for automotive applications and investigate the use of polarization to increase the contrast between fog and different targets for several fog types and wavelengths of light. This includes an analysis of the mechanism underlying contrast increase and a method to increase the contrast in the problematic case of a reflecting target. We take polarization and time-dependence into account and examine the ballistic, the single-scattered, the double-scattered, and the total detected light.

This work is structured as follows. Section 2 contains a description of the implemented Monte Carlo simulation (Section 2.1) and shows the analytic solutions of the vRTE for unobstructed propagation that were used to validate the simulation program (Section 2.2). In Section 3, we present results of the simulations. This includes unobstructed propagation (Section 3.1), perpendicularly illuminated targets (Section 3.2), and targets that are tilted with respect to the illumination direction (Section 3.3). Section 4 contains our conclusion.

## 2. Methods

### 2.1. Monte Carlo Simulation

In order to simulate light propagation in fog, we used a custom Monte Carlo method. Light propagation was modeled by tracing energy packets through the medium. Scattering was treated stochastically, and an optional obstacle was taken into account. The simulation program was implemented in OpenCL C 1.2, and calculations were carried out on GPUs.

The Monte Carlo simulation utilized the Stokes formalism to account for the light’s polarization. Descriptions of similar programs can be found in the literature [30,31,32,33,34,35]. Every energy packet is associated with a Stokes vector $I=\left(I,Q,U,V\right)$ and a local reference frame, where the conventions of Bohren and Huffman [36] were used, i.e., the propagation direction, and $e_{s}$ and $e_{p}$ form a right-handed orthogonal basis. The Stokes vector $I_{n}$ after *n* elastic scattering events depends on the previous one according to

(1a) $$\begin{array}{cc} I_{n}^{\prime } & =Z\left(\theta_{n}\right)R\left(\phi_{n}\right)I_{n-1}, \end{array}$$

(1b) $$\begin{array}{cc} I_{n} & =I_{n}^{\prime }\frac{I_{n-1}}{I_{n}^{\prime }}, \end{array}$$

with $\theta_{n}$ the scattering angle, $Z:\left[0,\pi \right]\to R^{4\times 4}$ the matrix-valued scattering function. $R\left(\phi_{n}\right)\in R^{4\times 4}$ is a rotation matrix that transforms Stokes vectors from the previous local frame into the one aligned to the scattering plane by rotating around the propagation direction by the angle $\phi_{n}$. Z(θ) consists of two 2×2 blocks on the diagonal for all θ due to the spherical symmetry of the droplets in fog [37]. A scattering angle of $\theta =0^{\circ }$, i.e., cosθ=1, corresponds to scattering along the original propagation direction. The normalization (1b) is necessary because the procedure described in the following represents an instance of importance sampling [30].

The probability density for the scattering angles [38] can be derived from Equation (1a). Expanding all expressions leads to

(2) $$\left(\begin{array}{c} I_{n}^{\prime }\\ Q_{n}^{\prime }\\ U_{n}^{\prime }\\ V_{n}^{\prime } \end{array}\right)=\left(\begin{array}{cccc} Z_{11}\left(\theta_{n}\right) & Z_{12}\left(\theta_{n}\right) & 0 & 0\\ Z_{21}\left(\theta_{n}\right) & Z_{22}\left(\theta_{n}\right) & 0 & 0\\ 0 & 0 & Z_{33}\left(\theta_{n}\right) & Z_{34}\left(\theta_{n}\right)\\ 0 & 0 & Z_{43}\left(\theta_{n}\right) & Z_{44}\left(\theta_{n}\right) \end{array}\right)\left(\begin{array}{cccc} 1 & 0 & 0 & 0\\ 0 & \cos(2\phi_{n}) & \sin(2\phi_{n}) & 0\\ 0 & -\sin(2\phi_{n}) & \cos(2\phi_{n}) & 0\\ 0 & 0 & 0 & 1 \end{array}\right)\left(\begin{array}{c} I_{n-1}\\ Q_{n-1}\\ U_{n-1}\\ V_{n-1} \end{array}\right).$$

By taking the probability distribution proportional to the ratio of $I_{n}^{\prime }$ and $I_{n-1}$, this results in

(3) $$p_{n}\left(\theta ,\phi \right)=N\frac{I_{n}^{\prime }}{I_{n-1}}=N\left(Z_{11}\left(\theta \right)+Z_{12}\left(\theta \right)\left(\frac{Q_{n-1}}{I_{n-1}}\cos\left(2\phi \right)+\frac{U_{n-1}}{I_{n-1}}\sin\left(2\phi \right)\right)\right)$$

with normalization constant $N={\left(2\pi \int_{0}^{\pi }Z_{11}\left(\theta^{\prime }\right)\sin\left(\theta^{\prime }\right)\;d\theta^{\prime }\right)}^{-1}$. The scattering angle $\theta_{n}$ is obtained from the marginalized probability distribution

(4) $$p_{n}\left(\theta \right)=\int_{0}^{2\pi }p_{n}\left(\theta ,\phi^{\prime }\right)\;d\phi^{\prime }=\frac{Z_{11}\left(\theta \right)}{\int_{0}^{\pi }Z_{11}\left(\theta^{\prime }\right)\sin\left(\theta^{\prime }\right)\;d\theta^{\prime }}$$

by means of inversion sampling. The conditional probability

(5) $$\begin{array}{cc} p_{n}\left(\phi |\theta_{n}\right) & =p_{n}\left(\theta_{n},\phi \right)/p_{n}\left(\theta_{n}\right)\\ \; & =\frac{1}{2\pi }\left[1+\frac{Z_{12}\left(\theta_{n}\right)}{Z_{11}\left(\theta_{n}\right)}\left(\frac{Q_{n-1}}{I_{n-1}}\cos\left(2\phi \right)+\frac{U_{n-1}}{I_{n-1}}\sin\left(2\phi \right)\right)\right] \end{array}$$

yields the rotation angle $\phi_{n}$ via rejection sampling. An exponential distribution [39] with expected value $1/\mu_{s}$ is used to sample the step sizes between scattering events.

The last flight method [40,41,42,43,44] is applied for variance reduction. If a scattering event takes place in the detector’s field of view (FOV), the energy packet contributes its Stokes vector to the total radiance, weighted with

(6) $$p_{n}\left(\theta ,\phi \right)e^{-\mu_{s}d_{n}}e^{-\mu_{a}\left(d_{n}+\ell_{n}\right)}/\left(d_{n}^{2}\Omega_{\det}\Delta t\right)$$

where θ and ϕ are the angles necessary to scatter toward the detector, $d_{n}$ the distance to the detector, $\ell_{n}$ the total path length up to the scattering event, $\Omega_{\det}$ the solid angle of the detector’s FOV, and Δt the size of a time bin. The detected time-dependent radiance is an average over the detector’s FOV and the time Δt.

**Figure 1:**
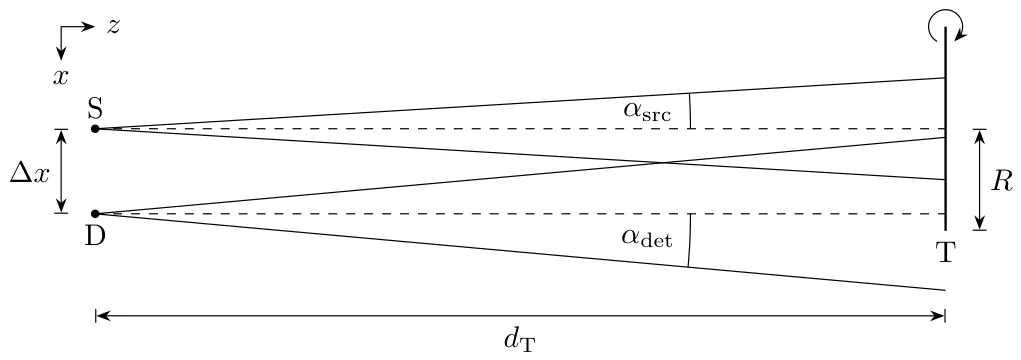
Schematic representation of the simulated bistatic lidar configuration. The point-like source (S) and detector (D) are separated by Δx, the source’s light cone has a half-angle of $\alpha_{\mathrm{src}}$, and the detector accepts light from a cone with half-angle $\alpha_{\det}$. The cones’ symmetry axes are parallel. Optionally, a disc-shaped target (T) of radius *R* is placed with its center on the source cone’s symmetry axis at the distance $d_{T}$ to the source. The target can be tilted around the axis through its center, which is parallel to the line from S to D.

We simulated a bistatic scanning lidar, as shown schematically in Figure 1. The light source and the detector are assumed to be point-like, separated by the distance Δx=2 cm, with the source emitting light into a cone of half-angle $\alpha_{\mathrm{src}}\geq 0$, and the detector accepting light from a cone of half-angle $\alpha_{\det}=0.1^{\circ }$. The symmetry axes of the emission and detection cones are aligned parallel to each other. In the detector’s local frame, $e_{p}=e_{x}$, and $e_{s}=e_{y}$, where $e_{x}$ and $e_{y}$ denote the unit vectors along the *x* and *y* directions. At time t=0, the source emits an infinitely short light pulse of wavelength λ=905 nm, λ=1460 nm or λ=1550 nm, where the first and the last one are common wavelengths for lidar systems [45], and the second one is interesting due to the larger water absorption at that wavelength. With $\alpha_{\det}$ given, the solid angle covered by the detector’s FOV is $\Omega_{\det}=2\pi \left(1-\cos\alpha_{\det}\right)$.

In order to simulate an obstacle in the beam path, a disc-shaped target of radius R=1 m can be placed with its center on the source cone’s symmetry axis at the distance $d_{T}$ to the source. This target can be tilted around an axis through its center, with the axis parallel to the line through source and detector. The detector facing side of the target has either a completely depolarizing Lambertian surface or a perfectly reflecting surface, and the far side is perfectly absorbing. Interactions with the target, even for a Lambertian surface, are not counted as scattering events within this study.

In our simulation, fog was modeled as an infinite homogeneous medium. Similarly to the distribution function proposed by Tampieri and Tomasi [46], the particle size distribution is given by

(7) $$n\left(r\right)=ar^{\alpha }\exp\left(-\frac{\alpha }{\gamma }{\left(\frac{r}{r_{c}}\right)}^{\gamma }\right),$$

where *r* is the water droplet radius, *a* a normalization constant such that $\int_{0}^{\infty }n\left(r\right)dr=1$, and $r_{c}$ the distribution’s mode. The full scattering function is obtained by integrating the Müller matrices of Mie scattering functions over the particle radius (from 0 to *∞*), weighted with the product of the scattering cross-section and the particle size distribution $C_{\mathrm{sca}}\left(r\right)n\left(r\right)$. Moreover, the scattering function Z is normalized such that

(8) $$2\pi \int_{0}^{\pi }Z_{11}\left(\theta \right)\sin\left(\theta \right)\;d\theta =1.$$

**Figure 2:**
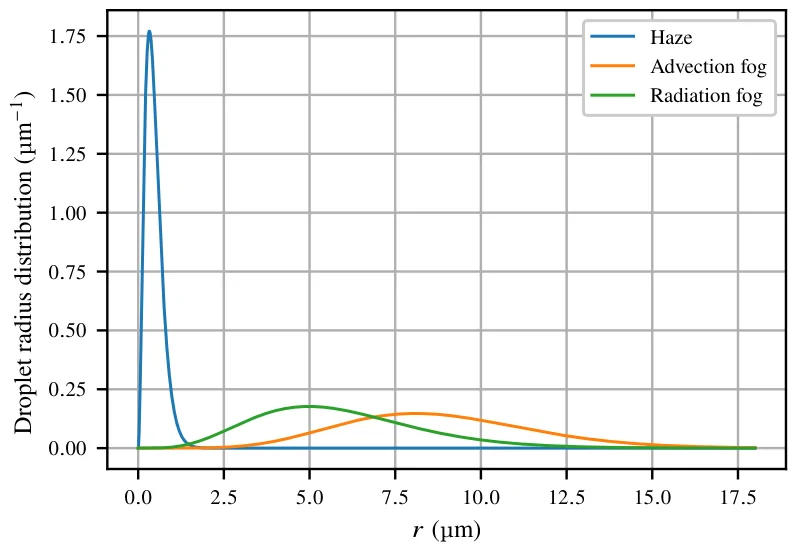
Size distribution of water droplets in haze, advection fog and radiation fog, resulting from Equation (7) and the parameters in Table 1.

**Figure 3:**
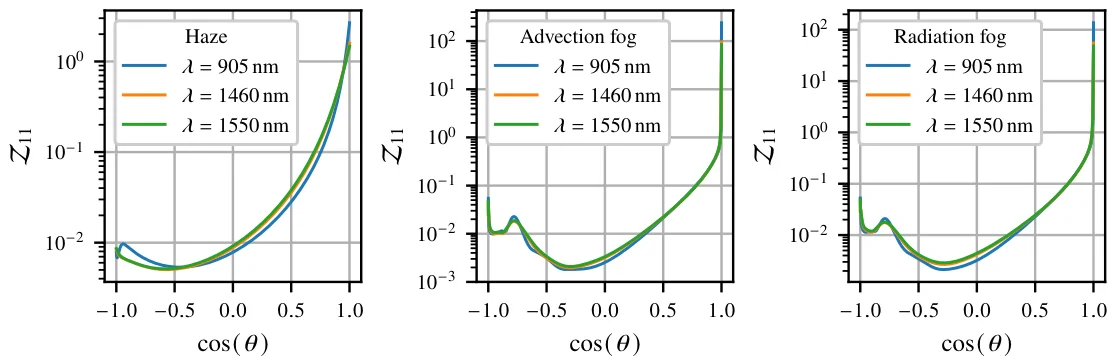
Upper left Müller matrix entries of the scattering functions for haze, advection fog and radiation fog at different wavelengths.

We consider three types of fog (haze, advection fog, and radiation fog), with their parameters for the particle size distribution (7) listed in Table 1. The parameters of these fog types are derived from the ones given by Tampieri and Tomasi [46] for “Haze 2”, “Radiation fog 2”, and “Advection fog 4”. In advection fog, the average droplet size is much larger than in haze. Figure 2 shows the resulting size distributions and Figure 3 the upper left entries of the scattering functions.

The wavelength dependence of scattering and absorption coefficients $\mu_{s}$ and $\mu_{a}$ is taken into account. They are chosen such that the visibility $v_{905}=3.9/\left(\mu_{s}\left(905\;nm\right)+\mu_{a}\left(905\;nm\right)\right)$ at a wavelength of 905 nm is 1000 m for haze, and 100 m for advection fog and radiation fog. The resulting scattering and absorption coefficients are given in Table 2. Even though the relative humidity in fog is close to 100%, the absorption due to water vapor is not taken into account. Its inclusion would not alter the results much; as for the wavelengths under consideration the absorption due to water vapor is similar to the absorption in droplets, and therefore scattering at the droplets dominates the light’s propagation.

In case a target is present, the distance between the light source and the disc’s center is chosen to be half the visibility at 905 nm, i.e., $d_{T}=v_{905}/2$. Hence, for haze the target is 500 m away, and for advection fog and radiation fog the target distance is 50 m. The length cΔt of light path length bins, with *c* the speed of light, is chosen as $\frac{1}{100}$ of the visibility at 905 nm, i.e., 10 m for haze and 1 m for advection fog and radiation fog.

### 2.2. Analytic vRTE Solutions

To validate the Monte Carlo simulation’s results, we compared them to analytic solutions of the vectorial radiative transfer equation. Green’s function $G_{1}\left(x,\hat{s},{\hat{s}}_{0},t\right)$ for the Stokes vector-valued radiance of single-scattered light from a unidirectional point source is given by [29]

(9) $$G_{1}\left(x,\hat{s},{\hat{s}}_{0},t\right)=c\mu_{s}e^{-\mu_{t}ct}Z\left(\arccos\left(\hat{s}\;\cdot \;{\hat{s}}_{0}\right)\right)R\left(\phi_{1}\right)S_{\mathrm{inc}}\int_{0}^{ct}\delta \left(x-\ell \hat{s}-\left(ct-\ell \right){\hat{s}}_{0}\right)\;d\ell ,\;t>0,$$

with x the position relative to the light source, $\hat{s}\in S^{2}$ the direction unit vector, ${\hat{s}}_{0}\in S^{2}$ and $S_{\mathrm{inc}}\in R^{4}$ the direction and Stokes vector of the source, and $\mu_{t}=\mu_{s}+\mu_{a}$. Furthermore, the Green’s function $G_{2}\left(x,\hat{s},{\hat{s}}_{0},t\right)$ for the radiance of double-scattered light from a unidirectional point source is [29]

(10) $$G_{2}\left(x,\hat{s},{\hat{s}}_{0},t\right)=2c\mu_{s}e^{-\mu_{t}ct}\int_{0}^{\ell^{*}}\frac{Z\left(\arccos\left(\hat{s}\;\cdot \;\hat{y}\right)\right)R\left(\phi_{2}\right)Z\left(\arccos\left(\hat{y}\;\cdot \;{\hat{s}}_{0}\right)\right)R\left(\phi_{1}\right)S_{\mathrm{inc}}}{|x-\ell \hat{s}-\left(ct-\ell \right){\hat{s}}_{0}{|}^{2}}\;d\ell$$

for t>|x|/c and

(11) $$\begin{array}{ccc} \hat{y} & \text{:}= & \frac{x-\ell \hat{s}-\left(ct-\ell \right){\hat{s}}_{0}+\tau^{*}{\hat{s}}_{0}}{\tau^{*}}, \end{array}$$

(12) $$\begin{array}{ccc} \tau^{*} & \text{:}= & \frac{1}{2}\frac{|x-\ell \hat{s}-\left(ct-\ell \right){\hat{s}}_{0}{|}^{2}}{ct-\ell -\left(x-\ell \hat{s}\right)\;\cdot \;{\hat{s}}_{0}}, \end{array}$$

(13) $$\begin{array}{ccc} \ell^{*} & \text{:}= & \frac{1}{2}\frac{{\left(ct\right)}^{2}-{\left|x\right|}^{2}}{ct-x\;\cdot \;\hat{s}}. \end{array}$$

Double exponential integration [47] is used to evaluate the integral in the double-scattered radiance Green’s function (10).

The aperture of the detector is included by integrating Green’s functions (9) and (10) with respect to $\hat{s}$. A cone source can be obtained by integration with respect to ${\hat{s}}_{0}$. For the single-scattered radiance, these numerical integrations are carried out via Gauss-Legendre quadrature. In order to evaluate the integrals over source and detector solid angles for the double-scattered radiance, Monte Carlo integration is utilized.

## 3. Results

In this section, we present results for the detected ballistic, single-scattered, double-scattered, and total radiance, obtained by means of the polarized Monte Carlo simulation for the bistatic lidar setup shown in Figure 1. Light emitted by the source is right-circularly polarized (RCP). The plots in this section show results for the unidirectional light source; results for the cone source are presented in the Supplementary Materials. Light path length axes in the plots are scaled by the visibility $v_{905}$ in the respective fog type at a wavelength of 905 nm. This causes the points when a signal is detected first to be not aligned for different fog types, but features generated by the targets appear at the same position. Table S1 in the Supplementary Materials lists the number of energy packets simulated for every parameter set.

### 3.1. Unobstructed Light Path

**Figure 4:**
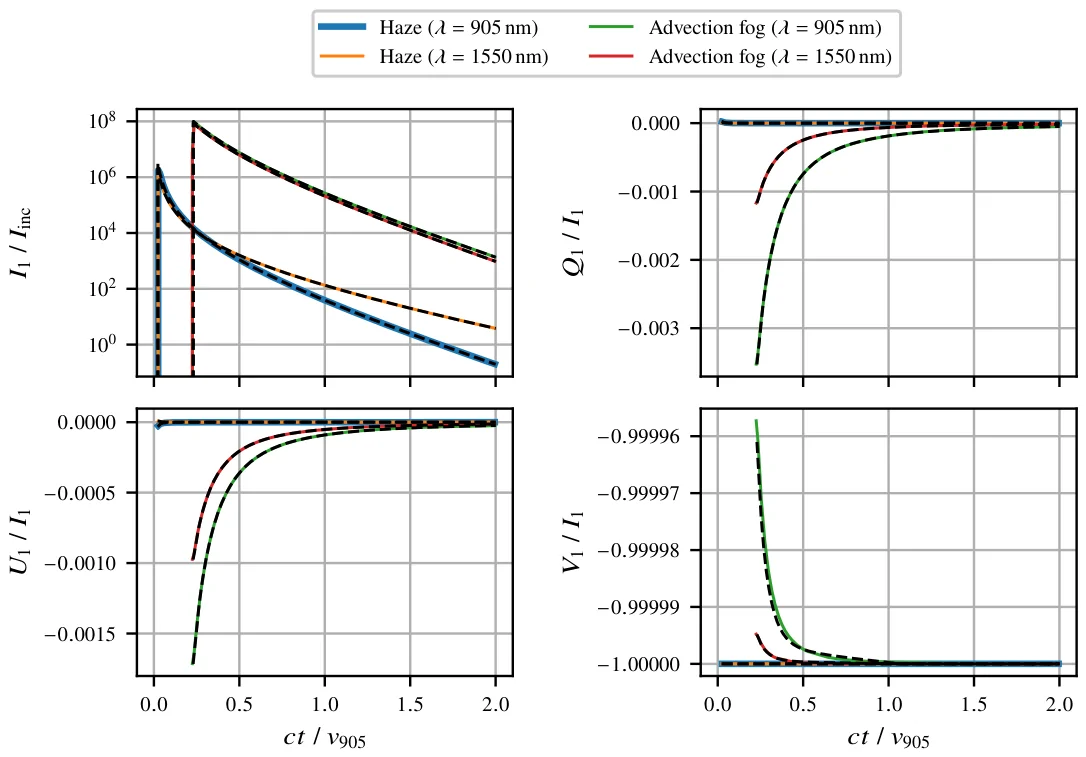
Stokes parameters $I_{1}$, $Q_{1}$, $U_{1}$, $V_{1}$ of the single-scattered radiance from a point-like unidirectional source. The radiance is shown for haze and advection fog at wavelengths of 905 nm and 1550 nm. Curves that would be hidden by other curves are drawn thicker. Analytic vRTE solutions are overlaid as dashed lines.

**Figure 5:**
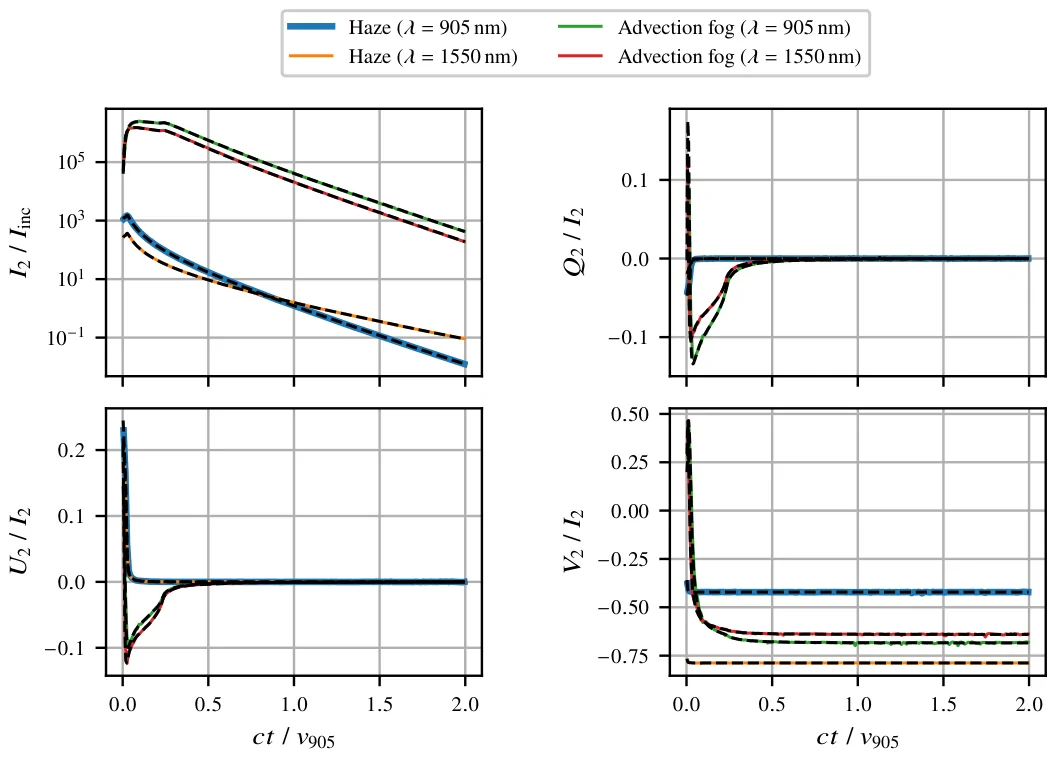
Stokes parameters $I_{2}$, $Q_{2}$, $U_{2}$, $V_{2}$ of the double-scattered radiance from a point-like unidirectional source. The radiance is shown for haze and advection fog at wavelengths of 905 nm and 1550 nm. Analytic vRTE solutions are overlaid as dashed lines.

In this section, we discuss the radiance in fog for an unobstructed light path. Figure 4 shows the Stokes parameters of the single-scattered radiance for a unidirectional light source and Figure 5 the double-scattered radiance. $I_{\mathrm{inc}}$ denotes the *I* component of $S_{\mathrm{inc}}$. Both for single- and double-scattered radiance, the simulation results agree very well with the numerically evaluated analytic vRTE solutions. Qualitatively, $I_{1}$ and $I_{2}$ exhibit a peak for all investigated fog types and wavelengths, followed by a decay. The specific shape of the peak depends on the scattering function Z, whereas the decay is mostly determined by the scattering coefficient $\mu_{s}$, with larger $\mu_{s}$ leading to a steeper decay (cf. Table 2). The source emits RCP light, yet in the medium single scattering close to the backwards direction at spherical droplets reverses the light’s handedness [48]. Hence, as can be seen from $V_{1}$ in Figure 4, the detected single-scattered light is almost exclusively left-circularly polarized (LCP). To a large extent, the double-scattered light is LCP and unpolarized, as indicated by the negative $V_{2}$ and the small $Q_{2}$ and $U_{2}$ in Figure 5. Nevertheless, the Stokes parameters $Q_{1}$, $Q_{2}$, $U_{1}$, and $U_{2}$ are non-zero, which is due to the offset between source and detector. The scattering matrix Z(θ) is diagonal for $\theta =180^{\circ }$ [37], and, due to continuity, Z(θ) is almost diagonal for scattering angles θ close to $180^{\circ }$. It should be noted that the angular region where Z(θ) is almost diagonal is relatively large in the NIR and SWIR range but much smaller in the visible spectrum. The small offset between source and detector in combination with the narrow beam and the small detector FOV means that the detected single-scattered light has been scattered by approximately $180^{\circ }$. Hence, Z(θ) is almost diagonal, and only small non-zero $Q_{1}$ and $U_{1}$ are introduced. Furthermore, the relative position and orientation of the source and detector and the finite detector FOV are the reasons why single-scattered light is detected only after some time because for detection, scattering events have to take place inside the detector’s FOV [29]. Shortly after emission of the light pulse, $Q_{2}$ and $U_{2}$ show deviations from zero due to short paths with scattering angles far from $0^{\circ }$ and $180^{\circ }$. For longer path lengths, $Q_{2}$ and $U_{2}$ are approximately zero since scattering happens mostly with angles near $0^{\circ }$ and $180^{\circ }$, and therefore the approximate diagonality of Z(θ) becomes relevant.

**Figure 6:**
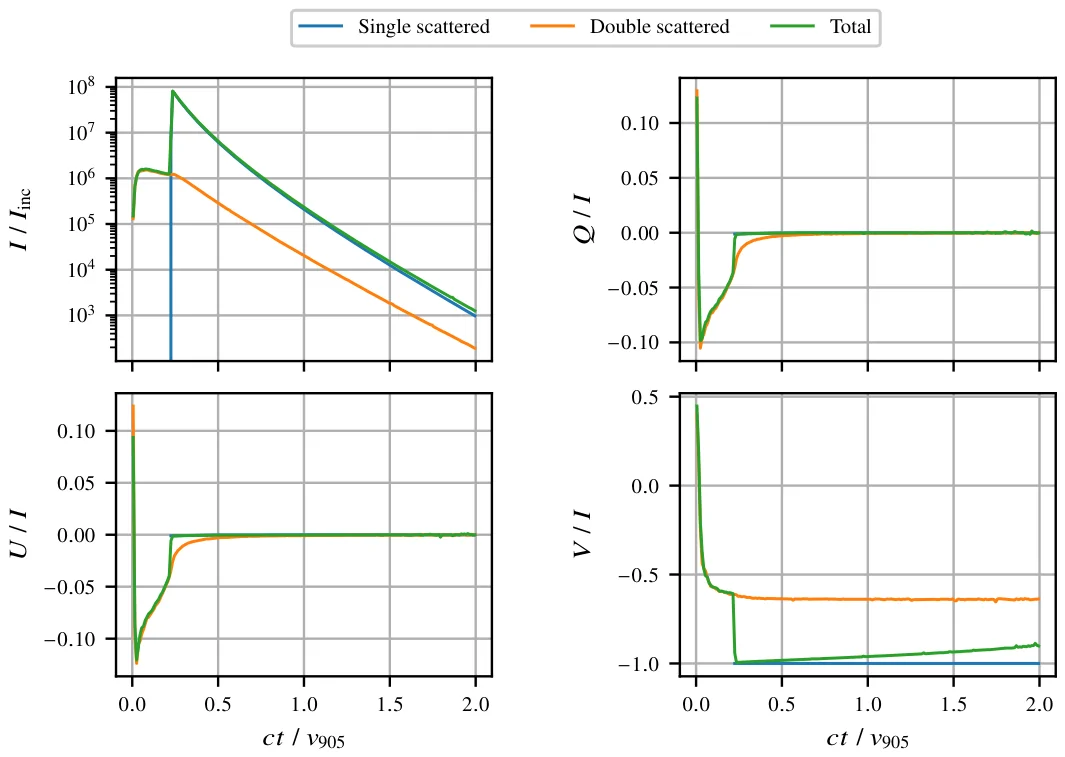
Stokes parameters of the single-scattered radiance, the double-scattered radiance, and the total radiance from a point-like unidirectional source. The radiance is shown for advection fog at a wavelength of 1550 nm.

Figure 6 shows the single-scattered, double-scattered, and total radiance for a unidirectional light source in advection fog at a wavelength of 1550 nm. Initially, double-scattered light contributes most to the total radiance. When the detection of single-scattered light becomes geometrically feasible, single scattering provides most of the radiance. The probability of light having undergone multiple scattering events increases with the path length, which leads to a decrease in the fraction that single- and double-scattered light contribute to the total radiance. The light’s polarization follows the polarization of its main constituent. Multiple scattering events lead to depolarization, which is evident in the *V* component.

Similar results for light propagation with an unobstructed path are obtained for a cone source of half-angle $\alpha_{\mathrm{src}}=0.1^{\circ }$. The corresponding radiance plots are shown in the Supplementary Materials in Figures S1–S3. The emission of light in a solid angle instead of a single direction leads to smoother radiance curves. As is the case for the unidirectional source, Monte Carlo simulation results and analytic vRTE solutions coincide very well.

### 3.2. Perpendicularly Illuminated Targets

In the next step, a disc target is inserted into the beam path (cf. Figure 1), oriented such that the emission direction of the source, or the symmetry axis of the source cone, is perpendicular to the target.

With the target present, it is possible to detect light which has not been scattered in the surrounding medium. The radiance $I_{0}$ from ballistically returned light forms a narrow peak at a time point that approximately corresponds to twice the target distance, which is due to the detector being located close to the light source. In Table 3, the time-integrated Stokes parameter $\int_{0}^{\infty }I_{0}\left(t\right)\;dt$ from a target with Lambertian surface is shown for all fog types and wavelengths, with the light source being unidirectional. The amounts of detected ballistic light differ because the scattering and absorption coefficients depend on fog type and wavelength, with the influence of the scattering coefficient dominating. The remaining Stokes parameters $Q_{0}$, $U_{0}$, and $V_{0}$ are zero since the Lambertian target’s surface completely depolarizes the incident light. Compared to the unidirectional source, the cone source reduces $\int_{0}^{\infty }I_{0}\left(t\right)\;dt$ for a Lambertian target by 14.6% if the target is at a distance of 50 m, whereas a distance of 500 m leads to a reduction by 1.5% (cf. Table S2). The reduction occurs because part of the illuminated area on the target is outside the detector’s FOV. With the target closer, the ratio of the illuminated area within the FOV to the total illuminated area is smaller. In case of a perfectly reflecting target, $I_{0}$ is also zero because the perpendicularly incident light is reflected back directly toward the light source, not toward the detector.

**Figure 7:**
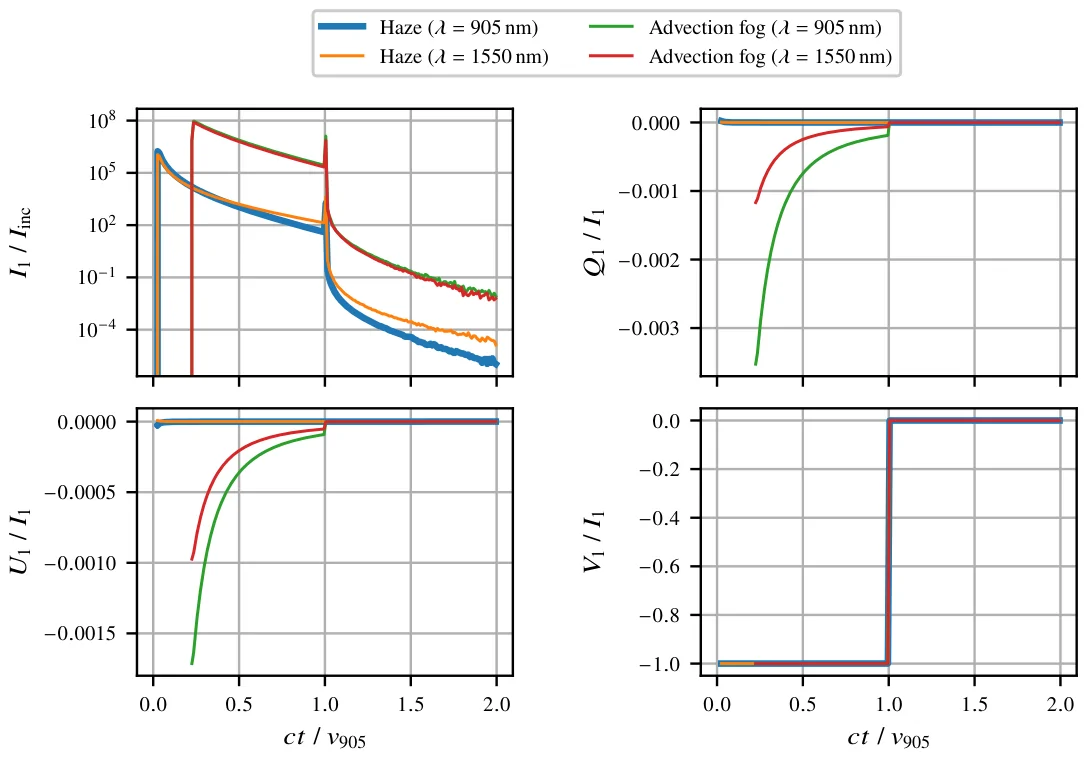
Like Figure 4, with a perpendicularly illuminated Lambertian disc target in the beam path. For $V_{1}$, all curves coincide.

**Figure 8:**
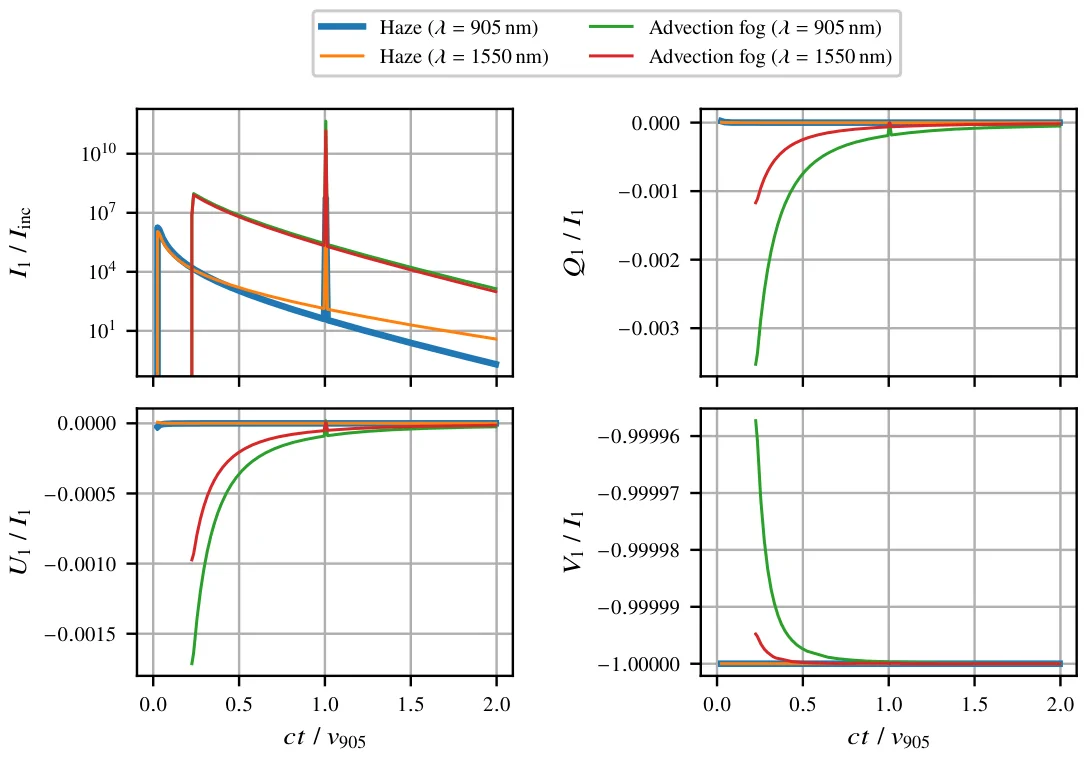
Like Figure 4, with a perpendicularly illuminated reflecting disc target in the beam path.

**Figure 9:**
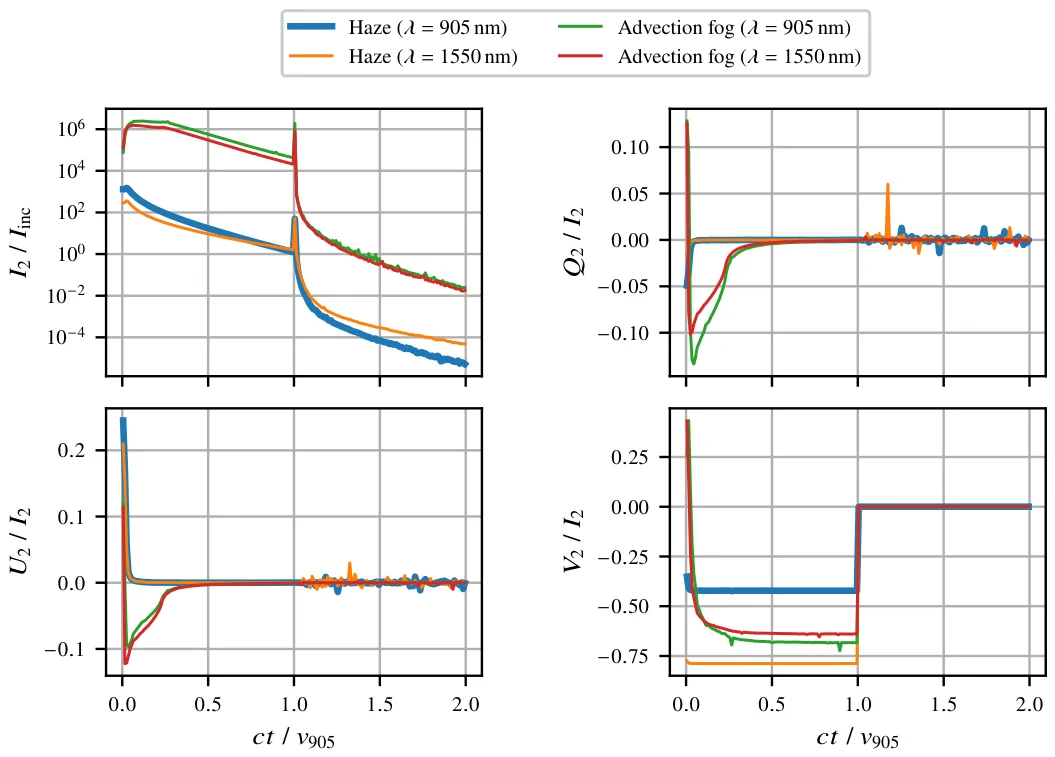
Like Figure 5, with a perpendicularly illuminated Lambertian disc target in the beam path.

**Figure 10:**
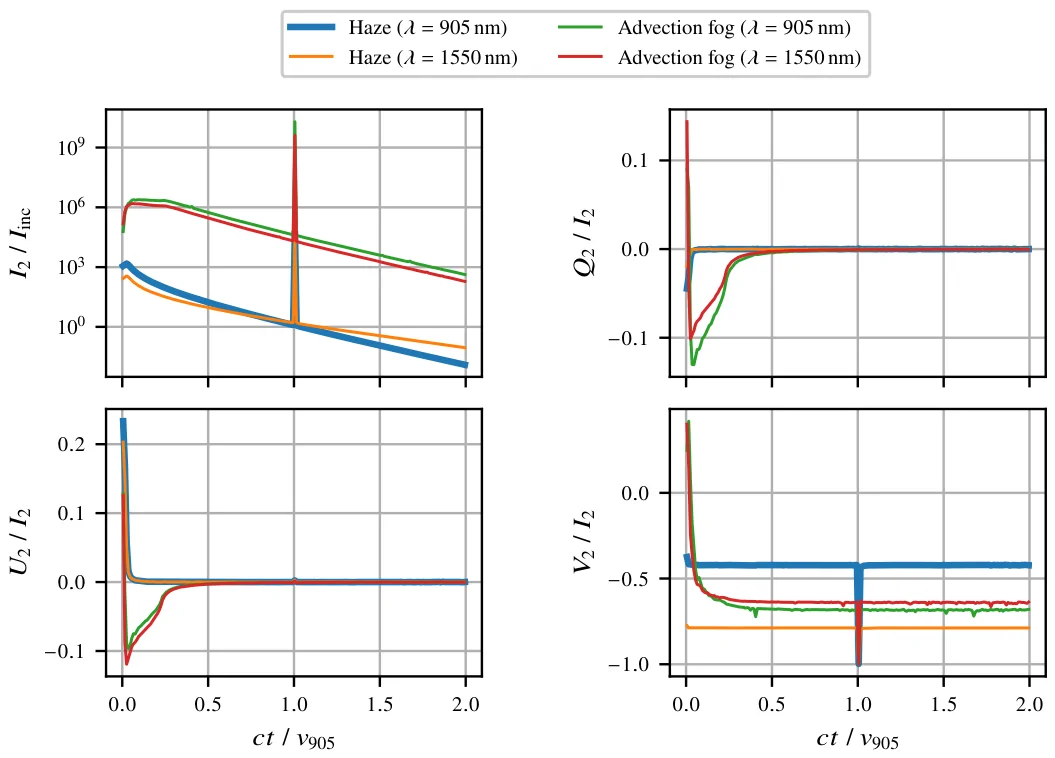
Like Figure 5, with a perpendicularly illuminated reflecting disc target in the beam path.

In the presence of a target, before light that has interacted with the target first reaches the detector, the radiance from single- and double-scattered light is the same as without the target. This holds both for single-scattered (cf. Figure 7 and Figure 8) and double-scattered light (cf. Figure 9 and Figure 10) and, due to the missing interaction with the target, is independent of the target’s surface properties. At the time corresponding to twice the target distance, the single- and double-scattered radiances $I_{1}$ and $I_{2}$ show a peak. In case of a reflecting target, the peaks are higher than for the Lambertian target. The reason for this is that the reflecting target keeps the incident unscattered light collimated when reflecting, whereas the Lambertian target distributes the light over a hemisphere. In addition, this relation leads to the single- and double-scattered radiance for the reflecting target to continue the overall trend after the target peak, which is due to paths that include a reflection at the target, then backscattering in the fog, and another reflection at the target. In contrast, for a Lambertian target, the radiance decays rapidly after the target peak. Exchanging the unidirectional source with a cone source yields similar results (cf. Figures S4–S7). Due to the divergent light beam, the curves for $I_{1}$ and $I_{2}$ are smoother and exhibit peaks of reduced height.

The target’s surface properties strongly influence the polarization of the detected light. It is assumed that the Lambertian surface depolarizes incident light completely. Therefore, as can be seen in Figure 7 and Figure 9, after the initial detection of mostly LCP light, $Q_{1}$, $Q_{2}$, $U_{1}$, $U_{2}$, $V_{1}$, and $V_{2}$ become zero after interaction with the target. In contrast, the perfectly reflecting surface only reverses the handedness of the polarization. Hence, the trends of $Q_{1}$, $Q_{2}$, $U_{1}$, $U_{2}$, $V_{1}$, and $V_{2}$ are continued after reflection at the target (cf. Figure 8 and Figure 10). Qualitatively, the cone source yields the same results.

**Figure 11:**
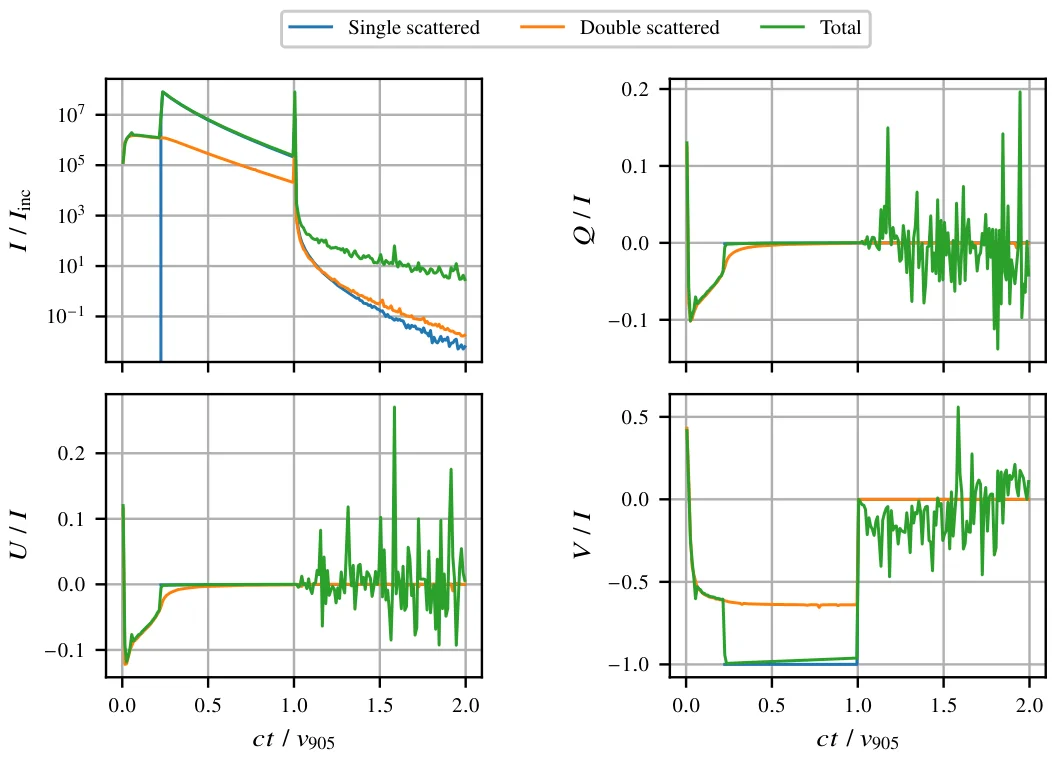
Like Figure 6, with a perpendicularly illuminated Lambertian disc target in the beam path.

**Figure 12:**
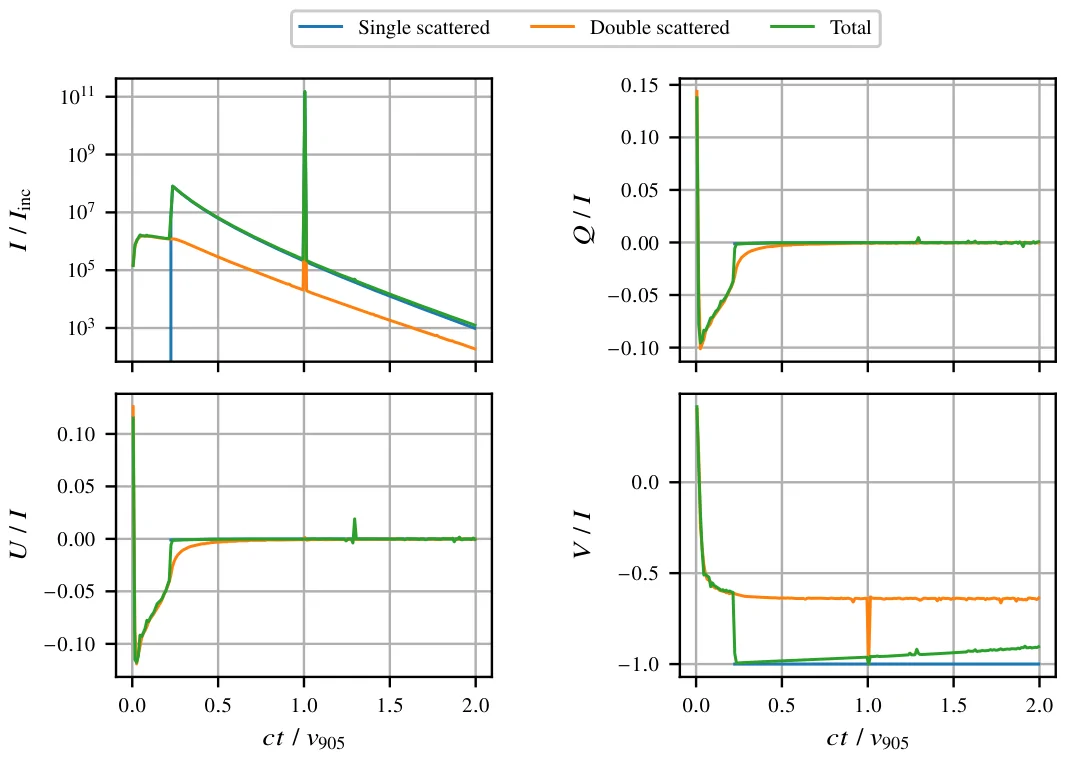
Like Figure 6, with a perpendicularly illuminated reflecting disc target in the beam path.

In Figure 11 and Figure 12, the single-scattered, the double-scattered, and the total radiance are shown for a unidirectional source and different targets in advection fog. The total radiance is the same as without a target for points in time that do not allow an interaction with the target due to its distance; cf. Figure 6. For the Lambertian target, single- and double scattering contribute only a small part to the total radiance after the target distance (cf. Figure 11). The reason is the backscattering of light into a hemisphere. Hence, most of the detected light has experienced further scattering events in the surrounding medium before detection. In contrast, for a reflecting target the total radiance consists still primarily of single- and double-scattered light after interaction with the target (cf. Figure 12). The reflecting surface conserves the collimated illumination, and therefore, the trends of the radiance are continued after the target, with a lower variance than in the case of the Lambertian target. The *V* component shows the increasing depolarization of the total radiance. A cone source leads to similar results both for the Lambertian target (cf. Figure S8) and the reflecting target (cf. Figure S9).

**Figure 13:**
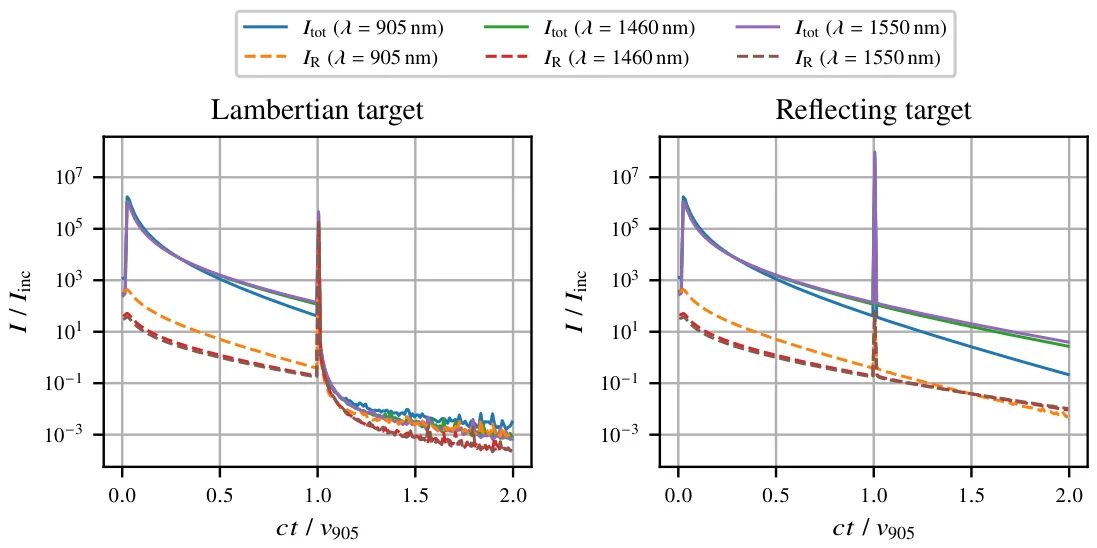
Total ($I_{\mathrm{tot}}$) and RCP radiance ($I_{R}$) from a RCP unidirectional source with perpendicularly illuminated Lambertian (**left**) and reflecting (**right**) targets. The radiance is shown for haze at wavelengths of 905 nm, 1460 nm, and 1550 nm.

**Figure 14:**
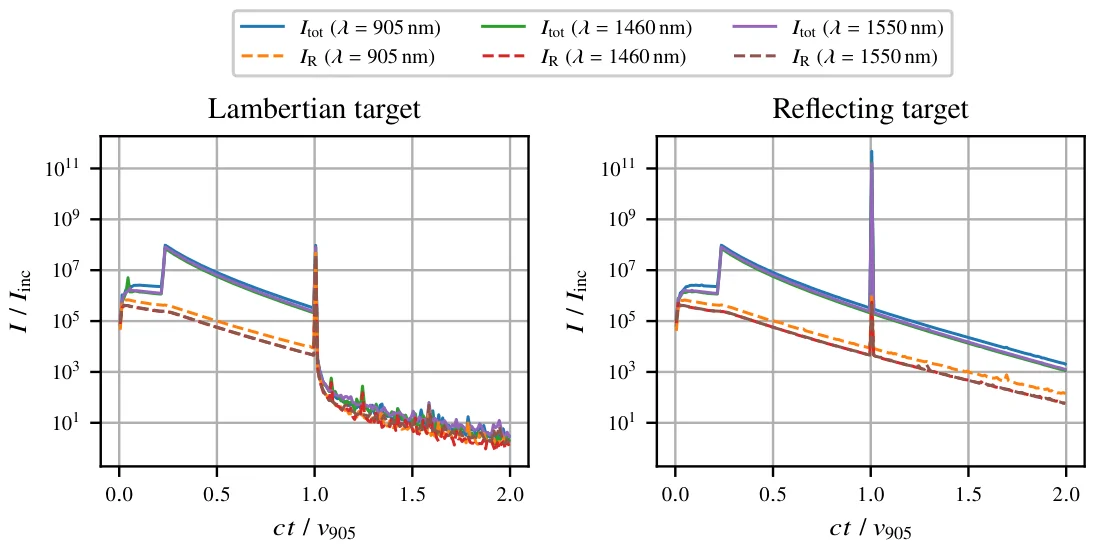
Like Figure 13, with advection fog.

**Figure 15:**
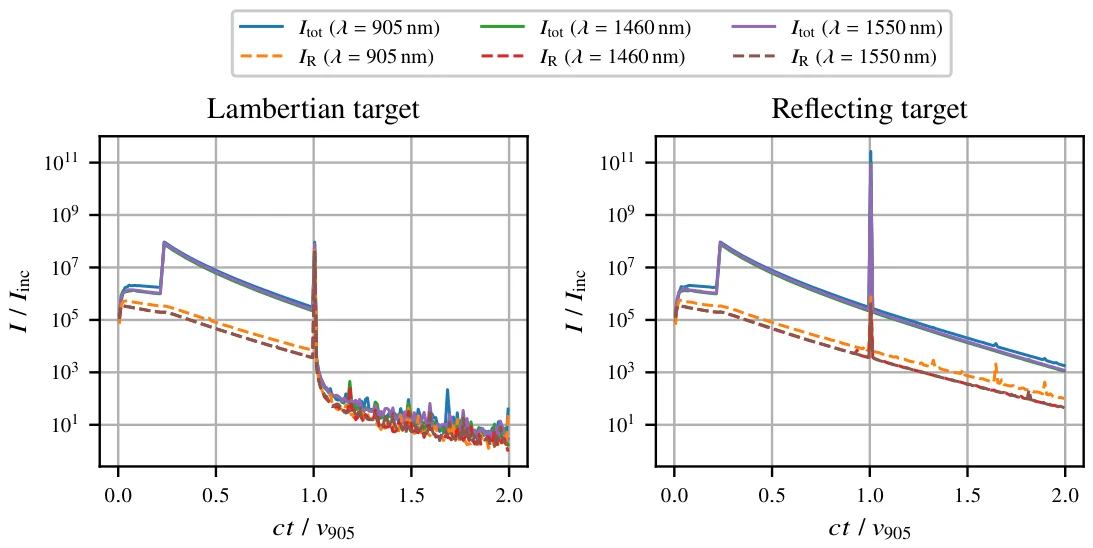
Like Figure 13, with radiation fog.

**Figure 16:**
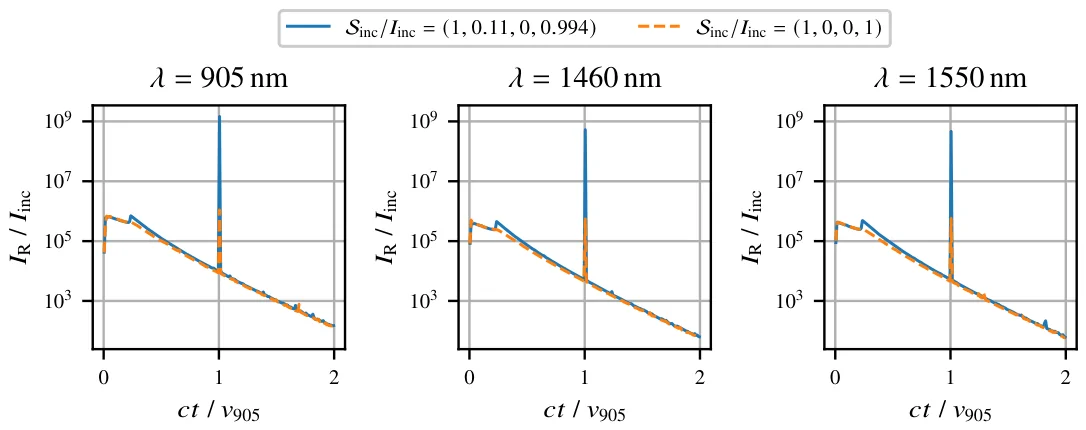
RCP radiance ($I_{R}$) from a unidirectional RCP (dashed orange line) or elliptically polarized source (solid blue line) with a perpendicularly illuminated reflecting target. The radiance is shown for advection fog at wavelengths of 905 nm, 1460 nm, and 1550 nm.

The differing behavior of the surface types has notable consequences for the polarization-based enhancement of contrast between the medium and target. The mechanism for increasing the contrast is to block more light from the fog backscattering than from the target return. In Figure 13, Figure 14 and Figure 15, the total radiance $I_{\mathrm{tot}}$ and the RCP radiance $I_{R}=\frac{1}{2}\left(I_{\mathrm{tot}}+V_{\mathrm{tot}}\right)$ from a unidirectional source are shown for different targets in haze, advection fog, and radiation fog at different wavelengths. Similarly, in Figure S10, the total radiance $I_{\mathrm{tot}}$ and the RCP and LCP radiances $I_{R}$ and $I_{L}=\frac{1}{2}\left(I_{\mathrm{tot}}-V_{\mathrm{tot}}\right)$ from a cone source are shown for different targets in advection fog at a wavelength of 1550 nm. When the light source emits CP light and only light of the same polarization is detected, a large fraction of the single-scattered light from the surrounding scattering medium can be filtered out. In the presented simulations, approximately at the fog single scattering peak, minimum ratios of $I_{R}/I_{\mathrm{tot}}\approx 3.4\times 10^{-5}$ for haze, $I_{R}/I_{\mathrm{tot}}\approx 3.0\times 10^{-3}$ for advection fog, and $I_{R}/I_{\mathrm{tot}}\approx 2.3\times 10^{-3}$ for radiation fog are obtained. Directly before the target peak, the ratio is $1.3\times 10^{-3}≲I_{R}/I_{\mathrm{tot}}≲9.4\times 10^{-3}$ for haze, $1.9\times 10^{-2}≲I_{R}/I_{\mathrm{tot}}≲2.7\times 10^{-2}$ for advection fog, and $1.5\times 10^{-2}≲I_{R}/I_{\mathrm{tot}}≲2.3\times 10^{-2}$ for radiation fog. This is possible because for scattering angles close to $180^{\circ }$ the single-scattered light reverses its handedness. A Lambertian target depolarizes the incident light completely, and therefore only half of the signal from the target is blocked ($I_{R}/I_{\mathrm{tot}}\approx 0.5$ at $ct=2d_{T}$). In total, less light is detected, but the contrast between the signals from the medium and target is increased. This outcome is distinct from that of a perfectly reflecting target, which reverses the polarization’s handedness, similarly to the surrounding medium in the case of backwards scattering. Thus, light that has been reflected at the target is also mostly filtered out. At $ct=2d_{T}$, the ratio of the RCP radiance and the total radiance is $6.1\times 10^{-7}≲I_{R}/I_{\mathrm{tot}}≲1.0\times 10^{-6}$ for haze, $2.3\times 10^{-6}≲I_{R}/I_{\mathrm{tot}}≲3.8\times 10^{-6}$ for advection fog, and $2.9\times 10^{-6}≲I_{R}/I_{\mathrm{tot}}≲4.9\times 10^{-6}$ for radiation fog. Again, less light is detected, but, in addition, the contrast has not increased. This can be addressed by making the CP illumination slightly elliptical, while keeping the detection of CP light; cf. Figure 16. Most of the single-scattered light is still blocked, but the linear component in the polarization allows more light reflected from the target to be detected. Where CP light is filtered out, the linear fraction remains and contributes to $I_{R}$, thereby enhancing the contrast considerably. The increase in the detected fog backscattering is negligible, yet the target peak is increased by three orders of magnitude for the chosen illumination polarization. The small modification to the illumination polarization does not significantly degrade the good contrast for a depolarizing target. Notably, the increased absorption of water at a wavelength of 1460 nm (cf. Table 2) has only a minor effect on the resulting radiance because the absorption coefficient $\mu_{a}$ is much smaller than the scattering coefficient $\mu_{s}$. This is similar to the situation with the other two wavelengths under investigation.

A wider FOV and a broader source cone increase the influence of multiply scattered light [49], which makes the polarization filtering less effective. For linearly polarized illumination, the effect of blocking single-scattered light can be achieved by detecting light with the plane of polarization rotated by $90^{\circ }$ relative to the illumination’s plane of polarization. The reason for this is that scattering angles near $180^{\circ }$ do not alter the polarization plane, which is in contradistinction to the reversal of the handedness for CP light. Similar to the use of slightly elliptically polarized light for illumination, the contrast of reflecting targets can be increased by ensuring that the polarization planes of the illumination and detection are almost, but not exactly, perpendicular to each other.

To summarize, whether the choice of illumination and detection polarization leads to a contrast increase depends on the target’s surface properties. The issues with reflecting surfaces can be circumvented by a minor modification of the illumination or detection polarization.

Ballesta-Garcia et al. [18] and Tremblay [21] have reported similar observations in experiments. Ballesta-Garcia et al. [18] built a polarized lidar system and performed object detection tests in a fog chamber. Experimentally, they found that polarization can be used to block a large part of the fog backscattering. The investigated target materials were roughly characterized as metallic or partially depolarizing. For depolarizing objects, the target peak stayed large with suitable polarized detection, whereas for a metallic target the detection polarization did not yield a marked advantage. However, Ballesta-Garcia et al. [18] did not give an explanation of the underlying mechanism. Their observations are related to our findings for depolarizing Lambertian and perfectly reflecting targets. Tremblay [21] measured the contrast and detection range for different target materials. Likewise, an increase in contrast was found for suitable polarization but not necessarily an increase in the detection range. The investigated materials were characterized by their linear and circular depolarization ratios. Information on the fog properties was not given. Ballesta-Garcia and Royo [19] implemented a Monte Carlo simulation of a polarized lidar system. They found the polarization effective in suppressing the fog backscattering. In their simulations, a fixed particle size was used. The perpendicularly illuminated target, which they assumed to be infinitely large, acted as a perfect mirror with an additional direction-independent Müller matrix applied to the Stokes vector of the reflected light. Tremblay and Roy [20] also implemented a Monte Carlo simulation of a polarized lidar system, with the focus of their investigation on the connection between the degrees of linear and circular polarization. As the scattering medium they considered advection fog and radiation fog. The actual target and its interaction with light was not modeled in their simulation.

### 3.3. Tilted Targets

**Figure 17:**
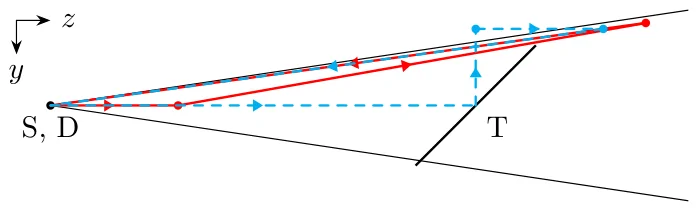
Simulated lidar configuration with a tilted target. The red path corresponds to double-scattered light without interaction with the target. The blue path includes reflection at the target, one scattering event by approximately $90^{\circ }$, and one scattering event by approximately $180^{\circ }$.

As most targets in automotive applications are not perpendicular to the illuminating beam, it is important to investigate the influence of tilted targets. In order to do so, we assumed the disc to be rotated by an angle of $45^{\circ }$ or $10^{\circ }$ around the axis through its center, which is parallel to the line through the light source and the detector; cf. Figure 17.

The radiance $I_{0}$ from the ballistically returned light exhibits a narrow peak for a Lambertian target surface. Since the surface depolarizes, the Stokes parameters $Q_{0}$, $U_{0}$, and $V_{0}$ are zero. The time-integrated Stokes parameter $\int_{0}^{\infty }I_{0}\left(t\right)\;dt$ is given in Table 4 for the unidirectional source and in Table S3 for the cone source. Tilting the target reduces $\int_{0}^{\infty }I_{0}\left(t\right)\;dt$ by a factor of $\cos(45^{\circ })$. The cone source leads to a reduction of $\int_{0}^{\infty }I_{0}\left(t\right)\;dt$ by approximately the same amount as observed for the perpendicularly illuminated target. With the perfectly reflecting target, no ballistic contribution is detected because at the target the light is not reflected toward the detector.

**Figure 18:**
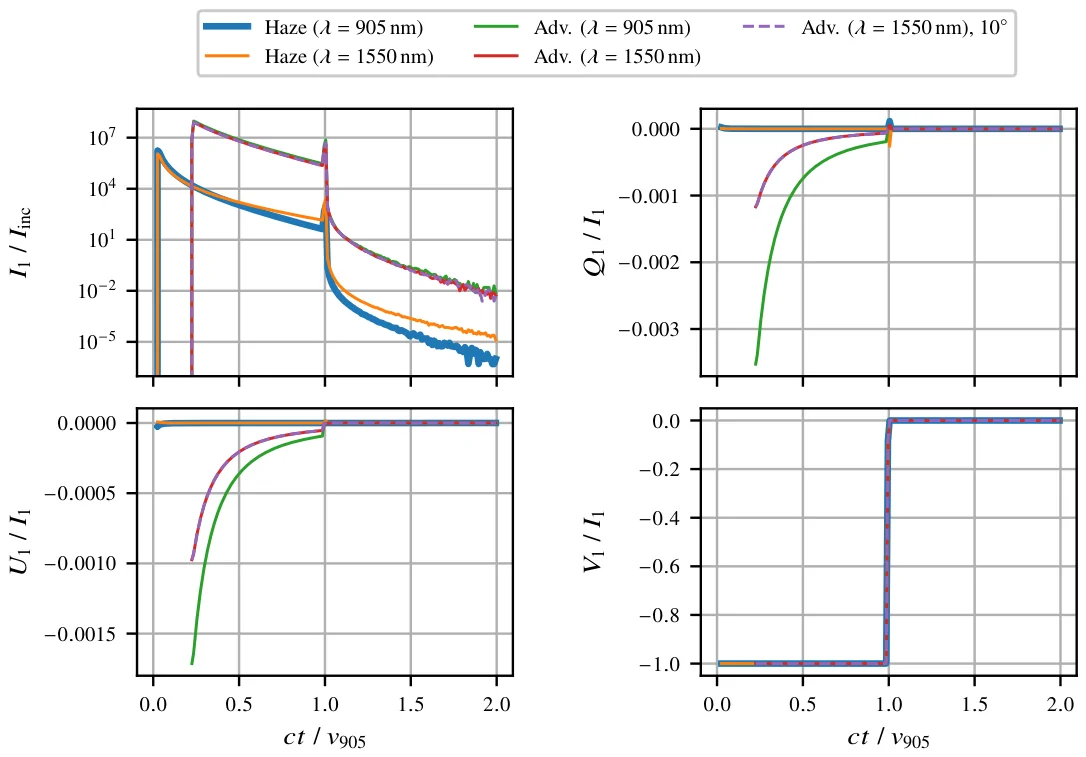
Like Figure 4, with a tilted ($45^{\circ }$, unless indicated otherwise) Lambertian disc target in the beam path. For $V_{1}$, all curves coincide.

**Figure 19:**
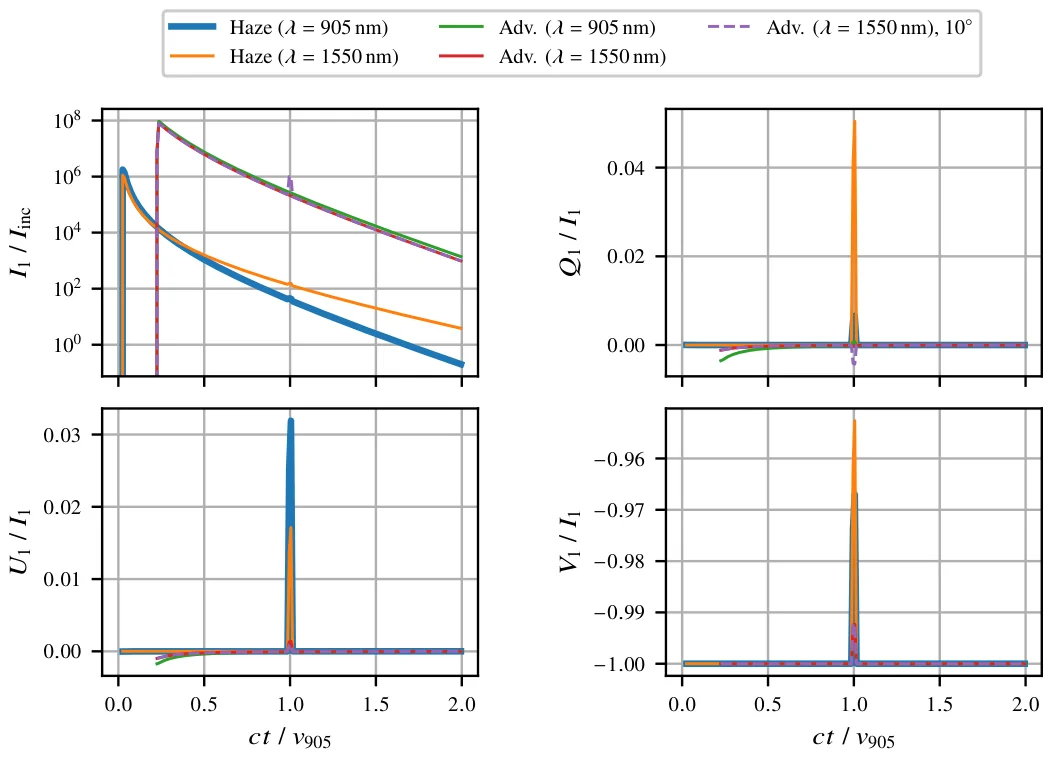
Like Figure 4, with a tilted ($45^{\circ }$, unless indicated otherwise) reflecting disc target in the beam path.

**Figure 20:**
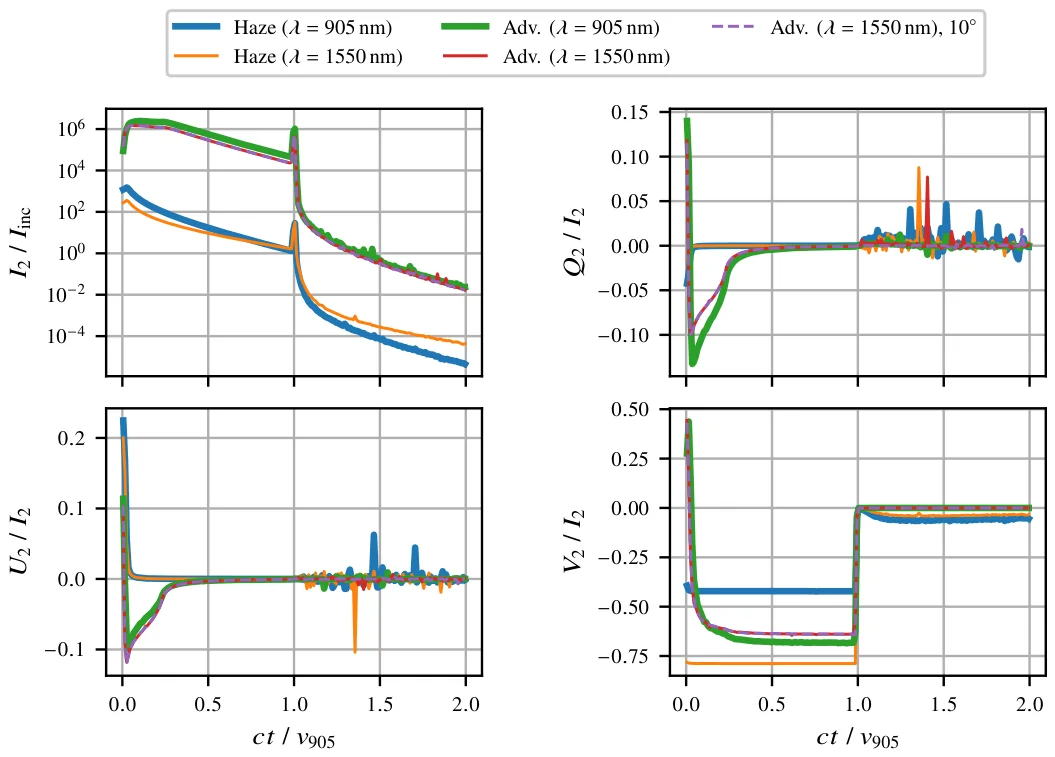
Like Figure 5, with a tilted ($45^{\circ }$, unless indicated otherwise) Lambertian disc target in the beam path.

**Figure 21:**
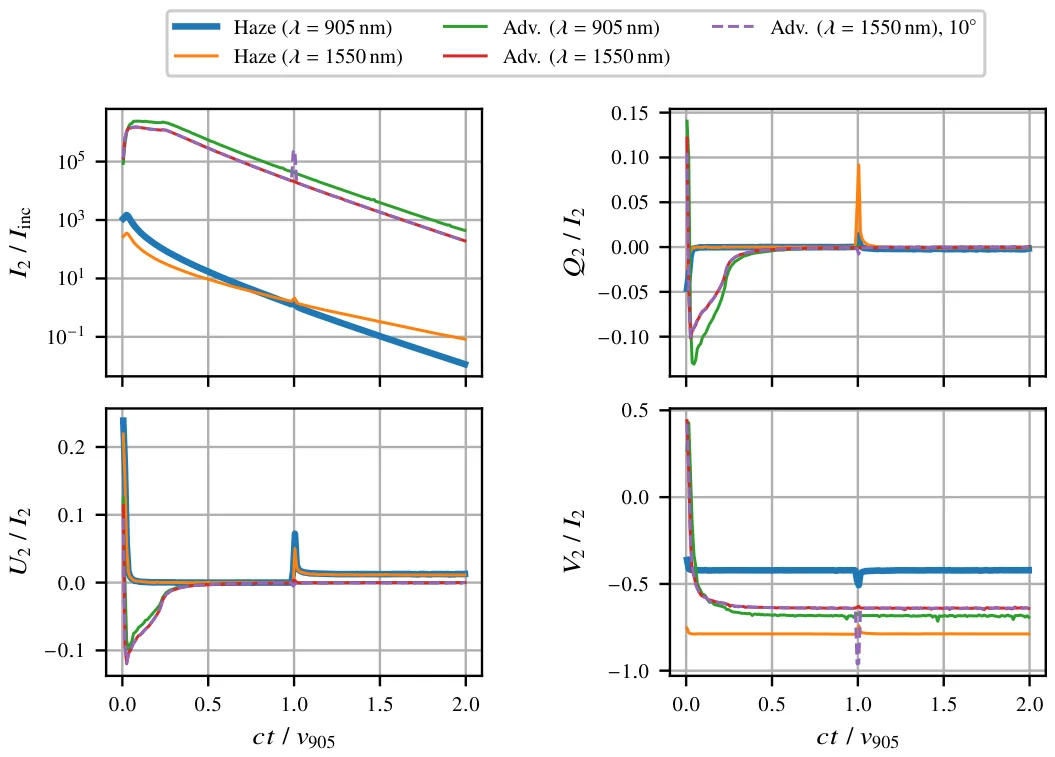
Like Figure 5, with a tilted ($45^{\circ }$, unless indicated otherwise) reflecting disc target in the beam path.

As can be seen in Figure 18, Figure 19, Figure 20 and Figure 21, the single- and double-scattered radiances $I_{1}$ and $I_{2}$ show a peak at the time corresponding to twice the target distance. Compared to the case of perpendicular illumination the peak heights are reduced, especially for the reflecting target. In case of a target that is tilted by $10^{\circ }$, the peak is larger than for a target tilted by $45^{\circ }$ and smaller than for perpendicular illumination. After the peak, $I_{1}$ and $I_{2}$ exhibit a decay that is very similar to the case of perpendicular illumination for the unidirectional source. The same observation can be made for the cone source as long as the beam cross-section is smaller than the target, i.e., for the target at a distance of 50 m (cf. Figures S11–S14). If a part of the beam misses the tilted target, as is the case for a target distance of 500 m, the medium behind the target influences the light propagation, and the detected radiance bears similarities to the case without a target. Hence, the detected radiance for $ct>2d_{T}$ is larger than in the case of perpendicular illumination. Qualitatively, the total radiance in the tilted case is very similar to that in the perpendicular case and therefore not shown.

Tilting the target has little effect on the detected polarization, provided the target covers the detector’s FOV and is larger than the beam cross-section.

For the Lambertian target, the polarization of single-scattered light is very similar to the case of perpendicular illumination (cf. Figure 18). The target depolarizes the light. If part of the light beam misses the target, as is possible for the cone source with the target at a distance of 500 m, the polarization is conserved (cf. Figure S11).

The polarization of the single-scattered radiance for the reflecting target is not substantially affected by tilting (cf. Figure 19), even if the light beam partly misses the target (cf. Figure S12). The reason for this is the polarization conserving the perfectly reflecting surface. Differences from the case of perpendicular illumination are due to the modified geometry and therefore different scattering angles and path lengths.

For advection fog with the Lambertian target, the polarization of the double-scattered light is very similar to the case of perpendicular illumination since the target covers the detector’s FOV completely (cf. Figure 20 and Figure S13). If the beam partly misses the target, i.e., for haze with the target in a distance of 500 m, $V_{2}$ is negative for $ct>2d_{T}$, even though the target acts depolarizing. This is due to the possibility of a first scattering event close to the source, which can deflect the light path such that part of the light misses the target. It is scattered a second time laterally offset behind the target and hence does not undergo complete depolarization (cf. Figure 17, red path). Haze in combination with the cone source additionally leads to non-zero $U_{2}$ and $Q_{2}$ for $ct>2d_{T}$ because a significant part of the light undergoes sidewards scattering processes.

The polarization of the double-scattered radiance for the reflecting target is very similar to the case of the perpendicularly illuminated target, apart from $U_{2}$, which is non-zero for $ct>2d_{T}$ (cf. Figure 21 and Figure S14). This feature can be attributed to paths that include at first a reflection at the target, then scattering by approximately $90^{\circ }$ roughly along the original propagation direction, and finally scattering by approximately $180^{\circ }$ toward the detector (cf. Figure 17, blue path). The non-zero $U_{2}$ is introduced by the $90^{\circ }$ scattering event.

Similar results are obtained with the cone source (cf. Figures S11–S14), but if part of the light beam misses the target, the changes in polarization can be more pronounced. The statements made in Section 3.2 regarding contrast enhancement by blocking single-scattered light still apply.

## 4. Conclusions

In this work, we presented a Monte Carlo simulation of a polarized bistatic scanning lidar system in an automotive context, utilizing the Stokes formalism. Light propagation in homogeneous haze, advection fog, and radiation fog in the presence of a target was investigated at wavelengths of 905 nm, 1460 nm, and 1550 nm. The scattering functions used were derived from Mie theory and physically plausible droplet size distributions.

The simulation was validated by the agreement of simulation results for unobstructed light propagation paths with analytic solutions of the vRTE for the single- and double-scattered radiance, with both a unidirectional and a cone source.

We implemented a disc-shaped target with a depolarizing Lambertian or perfectly reflecting surface. The target is either perpendicularly illuminated, or tilted; it is located half the visibility at a wavelength of 905 nm distant to the light source. For a depolarizing Lambertian surface, CP illumination, combined with a suitable detection polarization, allows the detected single-scattered light to be reduced by approximately two to four orders of magnitude, depending on the type of fog. Because half of the target signal is kept, this results in a large increase in contrast. A perfectly reflecting surface inverts the polarization’s handedness like backscattering in the surrounding fog does and hence does not result in a contrast increase. Yet we note that making the CP illumination slightly elliptical considerably increases the contrast also for the reflecting surface. A similar approach is possible with linear polarization. Choosing the illumination and detection polarization planes to be orthogonal to each other yields a contrast increase by approximately two orders of magnitude for depolarizing targets in advection fog and with almost orthogonal polarization planes also for reflecting targets. The influence of tilting the target by $45^{\circ }$ on the radiance is rather small, and the height of peaks that originate from the target is reduced. The fraction of ballistically returned light is reduced by a factor of $\cos(45^{\circ })$. Tilting the target by $10^{\circ }$ yields peak heights between those of tilting by $45^{\circ }$ and perpendicular illumination. There are small changes in the polarization due to the modified geometry and thereby altered light paths. The cone source tends to enlarge the differences from the case of perpendicular illumination.

As we took light at wavelengths of 905 nm and 1550 nm into account because these are common for lidar systems, and 1460 nm due to the large water absorption at this wavelength, it might be interesting to investigate other wavelengths. The wavelength dependence of the scattering and absorption coefficients might provide an optimum of these parameters that benefits lidar performance in fog and other adverse weather conditions.

## Figures and Tables

**Table 1:** Parameters for the particle size distribution (7) of haze, advection fog and radiation fog, derived from the parameters given by Tampieri and Tomasi [46] for “Haze 2”, “Advection fog 4” and “Radiation fog 2”.

	Haze	Advection Fog	Radiation Fog
α	2	6	4
γ	1.1	1.47	1.23
$r_{c}$	0.326 μm	8.1 μm	4.98 μm

**Table 2:** Scattering and absorption coefficients $\mu_{s}$ and $\mu_{a}$ for haze, advection fog and radiation fog at different wavelengths λ.

Fog Type	$\mu_{s}$ (m^−1^)	$\mu_{a}$ (m^−1^)
Haze (λ=905 nm)	3.900 × 10^−3^	1.553 × 10^−8^
Haze (λ=1460 nm)	2.387 × 10^−3^	6.296 × 10^−6^
Haze (λ=1550 nm)	2.169 × 10^−3^	2.183 × 10^−6^
Advection fog (λ=905 nm)	3.900 × 10^−2^	2.884 × 10^−6^
Advection fog (λ=1460 nm)	3.873 × 10^−2^	1.154 × 10^−3^
Advection fog (λ=1550 nm)	3.959 × 10^−2^	4.266 × 10^−4^
Radiation fog (λ=905 nm)	3.900 × 10^−2^	2.193 × 10^−6^
Radiation fog (λ=1460 nm)	3.924 × 10^−2^	8.836 × 10^−4^
Radiation fog (λ=1550 nm)	3.997 × 10^−2^	3.239 × 10^−4^

**Table 3:** Time-integrated Stokes parameter $\int_{0}^{\infty }I_{0}\left(t\right)\;dt$ of the ballistically returned radiance from a point-like unidirectional source with a perpendicularly illuminated Lambertian disc target in the beam path. The radiance is given for haze and advection fog at wavelengths of 905 nm and 1550 nm. $I_{0}$ exhibits a narrow peak at a time that corresponds to twice the target distance, whereas $Q_{0}$, $U_{0}$, and $V_{0}$ are zero due to the depolarizing target surface.

Fog Type	$\int_{0}^{\infty }I_{0}\left(t\right)\;dt$/($I_{\mathrm{inc}}\;\cdot \;1\;m/c$)
Haze (λ=905 nm)	8.074 × 10^5^
Haze (λ=1550 nm)	4.549 × 10^6^
Advection fog (λ=905 nm)	8.071 × 10^7^
Advection fog (λ=1550 nm)	7.293 × 10^7^

**Table 4:** Like Table 3, with a tilted ($45^{\circ }$) Lambertian disc target in the beam path.

Fog Type	$\int_{0}^{\infty }I_{0}\left(t\right)\;dt$/($I_{\mathrm{inc}}\;\cdot \;1\;m/c$)
Haze (λ=905 nm)	5.709 × 10^5^
Haze (λ=1550 nm)	3.216 × 10^6^
Advection fog (λ=905 nm)	5.707 × 10^7^
Advection fog (λ=1550 nm)	5.157 × 10^7^

## Data Availability

The raw data supporting the conclusions of this article will be available in Figshare at https://doi.org/10.6084/m9.figshare.32856092 after publication of this article.

## Supplementary Materials

The following supporting information can be downloaded at https://www.mdpi.com/article/10.3390/s26165053/s1, Table S1: Number of traced energy packets in the Monte Carlo simulations. RCP denotes right-circular polarization, EP elliptic polarization. L denotes a depolarizing Lambertian target, R a perfectly reflecting target; Table S2: Time-integrated Stokes parameter $\int_{0}^{\infty }I_{0}\left(t\right)\;dt$ of the ballistically returned radiance from a point-like cone source with a perpendicularly illuminated Lambertian disc target in the beam path. The radiance is given for haze and advection fog at wavelengths of 905 nm and 1550 nm. *I*_0_ exhibits a narrow peak at a time that corresponds to twice the target distance, whereas *Q*_0_, *U*_0_, and *V*_0_ are zero due to the depolarizing target surface; Table S3: Like Table S2, with a tilted ($45^{\circ }$) Lambertian disc target in the beam path; Figure S1: Stokes parameters *I*_1_, *Q*_1_, *U*_1_, *V*_1_ of the single-scattered radiance from a point-like cone source. The radiance is shown for haze and advection fog at wavelengths of 905 nm and 1550 nm. Curves that would be hidden by other curves are drawn thicker. Analytic vRTE solutions are overlaid as dashed lines; Figure S2: Stokes parameters *I*_2_, *Q*_2_, *U*_2_, *V*_2_ of the double-scattered radiance from a point-like cone source. The radiance is shown for haze and advection fog at wavelengths of 905 nm and 1550 nm. Analytic vRTE solutions are overlaid as dashed lines; Figure S3: Stokes parameters of the single-scattered radiance, the double-scattered radiance, and the total radiance from a point-like cone source. The radiance is shown for advection fog at a wavelength of 1550 nm; Figure S4: Like Figure S1, with a perpendicularly illuminated Lambertian disc target in the beam path. For *V*_1_, all curves coincide; Figure S5: Like Figure S1, with a perpendicularly illuminated reflecting disc target in the beam path; Figure S6: Like Figure S2, with a perpendicularly illuminated Lambertian disc target in the beam path; Figure S7: Like Figure S2, with a perpendicularly illuminated reflecting disc target in the beam path; Figure S8: Like Figure S3, with a perpendicularly illuminated Lambertian disc target in the beam path; Figure S9: Like Figure S3, with a perpendicularly illuminated reflecting disc target in the beam path; Figure S10: Total (*I*_tot_), right-circularly polarized (*I*_R_), and left-circularly polarized radiance (*I*_L_) from a right-circularly polarized cone source with perpendicularly illuminated Lambertian (left) and reflecting (right) targets. The radiance is shown for advection fog at a wavelength of 1550 nm; Figure S11: Like Figure S1, with a tilted Lambertian disc target in the beam path; Figure S12: Like Figure S1, with a tilted reflecting disc target in the beam path; Figure S13: Like Figure S2, with a tilted Lambertian disc target in the beam path; Figure S14: Like Figure S2, with a tilted reflecting disc target in the beam path.
