# Supplementary material for: Numeric Analysis of Polarized Lidar Contrast Enhancement Across Diverse Targets in Fog
Source: Sensors (Basel). 2026 Aug 9;26(16):5053. doi: 10.3390/s26165053 (PMC13517271; doi:10.3390/s26165053)
Supplement: Supplementary file 1 [file sensors-26-05053-s001.zip › sensors-4426918-supplementary.pdf]

Numeric analysis of polarized lidar contrast enhancement across diverse targets in fog: supplemental document

Manuel Petzi Dominik Reitzle Alwin Kienle

Abstract

This document contains plots and tables of the time-dependent radiance at the simulated detector in the bistatic lidar configuration described in the main document. The light source emits right-circularly polarized light into a cone of half-angle $\alpha_{\mathrm{src}} = 0.1°$.

Table S1 lists the number of energy packets traced in every simulation presented in the main document and this supplemental document.

Figures S1, S2, and S3 show the single scattered, the double scattered, and the total radiance for an unobstructed light path. The analytic solutions of the vectorial radiative transfer equation for single and double scattered light are overlaid on the Monte Carlo simulation results in Figures S1 and S2, showing very good agreement. The total radiance consists mostly of single scattered light and, when the detection of single scattered light is geometrically not possible, double scattered light. Since scattering at spherical droplets in backwards direction reverses the handedness of the light's polarization, the Stokes parameter V is negative.

The ballistically returned light, single scattered, double scattered, and total radiance for a perpendicularly illuminated Lambertian target are shown in Table S2, and in Figures S4, S6, and S8. Single scattered, double scattered, and total radiance for a perpendicularly illuminated perfectly reflecting target are shown in Figures S5, S7, and S9. The total radiance, the right-circularly polarized radiance, and the left-circularly polarized radiance for Lambertian and reflecting targets are shown in Figure S10. The target causes a peak to appear at a time that corresponds to twice the target distance. After interaction with the Lambertian target the light is mostly depolarized, whereas the reflecting target conserves the polarization.

For a Lambertian target that is tilted by 45°, the ballistic, the single scattered, and the double scattered radiance are given in Table S3, and in Figures S11 and S13. Furthermore, Figures S12 and S14 show the single and double scattered radiance for a tilted, perfectly reflecting target. Tilting the target leads to a reduced peak height. In case that part of the beam misses the depolarizing target, the polarization is conserved after the target peak. Tilting causes no significant changes for the reflecting target.

Table S1. Number of traced energy packets in the Monte Carlo simulations. RCP denotes right-circular polarization, EP elliptic polarization. L denotes a depolarizing Lambertian target, R a perfectly reflecting target.

Fog type	λ (nm)	Polarization	α_{src} (°)	Scatt. order	Target	No. of packets
Haze	905	RCP	0	1	no	1.0×10^{11}
Haze	1550	RCP	0	1	no	1.0×10^{11}
Advection	905	RCP	0	1	no	1.0×10^{11}
Advection	1550	RCP	0	1	no	1.0×10^{11}
Haze	905	RCP	0	2	no	1.6×10^{12}
Haze	1550	RCP	0	2	no	1.6×10^{12}
Advection	905	RCP	0	2	no	1.6×10^{12}
Advection	1550	RCP	0	2	no	1.6×10^{12}
Haze	905	RCP	0.1	1	no	1.0×10^{11}
Haze	1550	RCP	0.1	1	no	1.0×10^{11}
Advection	905	RCP	0.1	1	no	1.0×10^{11}
Advection	1550	RCP	0.1	1	no	1.0×10^{11}
Haze	905	RCP	0.1	2	no	1.6×10^{12}
Haze	1550	RCP	0.1	2	no	1.6×10^{12}

Continued on next page

Table S1. Number of traced energy packets in the Monte Carlo simulations. RCP denotes right-circular polarization, EP elliptic polarization. L denotes a depolarizing Lambertian target, R a perfectly reflecting target.

Fog type	λ (nm)	Polarization	α_{src} (°)	Scatt. order	Target	No. of packets
Advection	905	RCP	0.1	2	no	1.6×10^{12}
Advection	1550	RCP	0.1	2	no	1.6×10^{12}
Haze	905	RCP	0	0	L perp.	1.6×10^{12}
Haze	1550	RCP	0	0	L perp.	1.6×10^{12}
Advection	905	RCP	0	0	L perp.	1.6×10^{12}
Advection	1550	RCP	0	0	L perp.	1.6×10^{12}
Haze	905	RCP	0	1	L perp.	3.2×10^{12}
Haze	1550	RCP	0	1	L perp.	8.0×10^{11}
Advection	905	RCP	0	1	L perp.	4.0×10^{11}
Advection	1550	RCP	0	1	L perp.	4.0×10^{11}
Haze	905	RCP	0	2	L perp.	3.2×10^{12}
Haze	1550	RCP	0	2	L perp.	1.3×10^{13}
Advection	905	RCP	0	2	L perp.	6.4×10^{12}
Advection	1550	RCP	0	2	L perp.	6.4×10^{12}
Haze	905	RCP	0.1	0	L perp.	1.6×10^{12}
Haze	1550	RCP	0.1	0	L perp.	1.6×10^{12}
Advection	905	RCP	0.1	0	L perp.	1.6×10^{12}
Advection	1550	RCP	0.1	0	L perp.	1.6×10^{12}
Haze	905	RCP	0.1	1	L perp.	3.2×10^{12}
Haze	1550	RCP	0.1	1	L perp.	8.0×10^{11}
Advection	905	RCP	0.1	1	L perp.	1.0×10^{11}
Advection	1550	RCP	0.1	1	L perp.	4.0×10^{11}
Haze	905	RCP	0.1	2	L perp.	3.2×10^{12}
Haze	1550	RCP	0.1	2	L perp.	1.3×10^{13}
Advection	905	RCP	0.1	2	L perp.	6.4×10^{12}
Advection	1550	RCP	0.1	2	L perp.	1.6×10^{12}
Haze	905	RCP	0	1	R perp.	1.0×10^{11}
Haze	1550	RCP	0	1	R perp.	1.0×10^{11}
Advection	905	RCP	0	1	R perp.	1.0×10^{11}
Advection	1550	RCP	0	1	R perp.	1.0×10^{11}
Haze	905	RCP	0	2	R perp.	1.6×10^{12}
Haze	1550	RCP	0	2	R perp.	1.6×10^{12}
Advection	905	RCP	0	2	R perp.	1.6×10^{12}
Advection	1550	RCP	0	2	R perp.	1.6×10^{12}
Haze	905	RCP	0.1	1	R perp.	1.0×10^{11}
Haze	1550	RCP	0.1	1	R perp.	1.0×10^{11}
Advection	905	RCP	0.1	1	R perp.	1.0×10^{11}
Advection	1550	RCP	0.1	1	R perp.	1.0×10^{11}
Haze	905	RCP	0.1	2	R perp.	1.6×10^{12}
Haze	1550	RCP	0.1	2	R perp.	1.6×10^{12}
Advection	905	RCP	0.1	2	R perp.	1.6×10^{12}
Advection	1550	RCP	0.1	2	R perp.	6.4×10^{12}
Haze	905	RCP	0	0	L tilted (45°)	1.6×10^{12}
Haze	1550	RCP	0	0	L tilted (45°)	1.6×10^{12}
Advection	905	RCP	0	0	L tilted (45°)	1.6×10^{12}
Advection	1550	RCP	0	0	L tilted (45°)	1.6×10^{12}
Haze	905	RCP	0	1	L tilted (45°)	8.0×10^{11}
Haze	1550	RCP	0	1	L tilted (45°)	8.0×10^{11}
Advection	905	RCP	0	1	L tilted (45°)	1.0×10^{11}
Advection	1550	RCP	0	1	L tilted (45°)	4.0×10^{11}
Haze	905	RCP	0	2	L tilted (45°)	1.3×10^{13}

Continued on next page

Table S1. Number of traced energy packets in the Monte Carlo simulations. RCP denotes right-circular polarization, EP elliptic polarization. L denotes a depolarizing Lambertian target, R a perfectly reflecting target.

Fog type	λ (nm)	Polarization	α_{src} (°)	Scatt. order	Target	No. of packets
Haze	1550	RCP	0	2	L tilted (45°)	1.3×10^{13}
Advection	905	RCP	0	2	L tilted (45°)	6.4×10^{12}
Advection	1550	RCP	0	2	L tilted (45°)	6.4×10^{12}
Haze	905	RCP	0.1	0	L tilted (45°)	1.6×10^{12}
Haze	1550	RCP	0.1	0	L tilted (45°)	1.6×10^{12}
Advection	905	RCP	0.1	0	L tilted (45°)	1.6×10^{12}
Advection	1550	RCP	0.1	0	L tilted (45°)	1.6×10^{12}
Haze	905	RCP	0.1	1	L tilted (45°)	8.0×10^{11}
Haze	1550	RCP	0.1	1	L tilted (45°)	8.0×10^{11}
Advection	905	RCP	0.1	1	L tilted (45°)	4.0×10^{11}
Advection	1550	RCP	0.1	1	L tilted (45°)	4.0×10^{11}
Haze	905	RCP	0.1	2	L tilted (45°)	3.2×10^{12}
Haze	1550	RCP	0.1	2	L tilted (45°)	3.2×10^{12}
Advection	905	RCP	0.1	2	L tilted (45°)	6.4×10^{12}
Advection	1550	RCP	0.1	2	L tilted (45°)	6.4×10^{12}
Haze	905	RCP	0	1	R tilted (45°)	1.0×10^{11}
Haze	1550	RCP	0	1	R tilted (45°)	1.0×10^{11}
Advection	905	RCP	0	1	R tilted (45°)	1.0×10^{11}
Advection	1550	RCP	0	1	R tilted (45°)	1.0×10^{11}
Haze	905	RCP	0	2	R tilted (45°)	1.6×10^{12}
Haze	1550	RCP	0	2	R tilted (45°)	1.6×10^{12}
Advection	905	RCP	0	2	R tilted (45°)	1.6×10^{12}
Advection	1550	RCP	0	2	R tilted (45°)	1.6×10^{12}
Haze	905	RCP	0.1	1	R tilted (45°)	1.0×10^{11}
Haze	1550	RCP	0.1	1	R tilted (45°)	1.0×10^{11}
Advection	905	RCP	0.1	1	R tilted (45°)	1.0×10^{11}
Advection	1550	RCP	0.1	1	R tilted (45°)	1.0×10^{11}
Haze	905	RCP	0.1	2	R tilted (45°)	1.6×10^{12}
Haze	1550	RCP	0.1	2	R tilted (45°)	1.6×10^{12}
Advection	905	RCP	0.1	2	R tilted (45°)	1.6×10^{12}
Advection	1550	RCP	0.1	2	R tilted (45°)	6.4×10^{12}
Advection	1550	RCP	0	$0-\infty$	no	4.0×10^{12}
Advection	1550	RCP	0.1	$0-\infty$	no	1.0×10^{12}
Advection	1550	RCP	0	$0-\infty$	L perp.	4.0×10^{12}
Advection	1550	RCP	0.1	$0-\infty$	L perp.	2.8×10^{12}
Advection	1550	RCP	0	$0-\infty$	R perp.	7.0×10^{11}
Advection	1550	RCP	0.1	$0-\infty$	R perp.	7.0×10^{11}
Advection	1550	RCP	0	1	L tilted (10°)	4.0×10^{11}
Advection	1550	RCP	0	1	R tilted (10°)	4.0×10^{11}
Advection	1550	RCP	0	2	L tilted (10°)	1.3×10^{13}
Advection	1550	RCP	0	2	R tilted (10°)	6.4×10^{12}
Advection	905	EP	0	$0-\infty$	R perp.	7.0×10^{11}
Advection	1460	EP	0	$0-\infty$	R perp.	7.0×10^{11}
Advection	1550	EP	0	$0-\infty$	R perp.	7.0×10^{11}
Advection	905	RCP	0	$0-\infty$	L perp.	4.0×10^{12}
Advection	905	RCP	0	$0-\infty$	R perp.	5.0×10^{11}
Advection	1460	RCP	0	$0-\infty$	L perp.	1.0×10^{12}
Advection	1460	RCP	0	$0-\infty$	R perp.	5.0×10^{11}
Haze	905	RCP	0	$0-\infty$	L perp.	1.0×10^{12}
Haze	905	RCP	0	$0-\infty$	R perp.	5.0×10^{11}
Haze	1460	RCP	0	$0-\infty$	L perp.	1.0×10^{12}

Continued on next page

Table S1. Number of traced energy packets in the Monte Carlo simulations. RCP denotes right-circular polarization, EP elliptic polarization. L denotes a depolarizing Lambertian target, R a perfectly reflecting target.

Fog type	λ (nm)	Polarization	α_{src} (°)	Scatt. order	Target	No. of packets
Haze	1460	RCP	0	$0-\infty$	R perp.	5.0×10^{11}
Haze	1550	RCP	0	$0-\infty$	L perp.	7.0×10^{11}
Haze	1550	RCP	0	$0-\infty$	R perp.	5.0×10^{11}
Radiation	905	RCP	0	$0-\infty$	L perp.	7.0×10^{11}
Radiation	905	RCP	0	$0-\infty$	R perp.	5.0×10^{11}
Radiation	1460	RCP	0	$0-\infty$	L perp.	7.0×10^{11}
Radiation	1460	RCP	0	$0-\infty$	R perp.	5.0×10^{11}
Radiation	1550	RCP	0	$0-\infty$	R perp.	5.0×10^{11}
Radiation	1550	RCP	0	$0-\infty$	L perp.	7.0×10^{11}

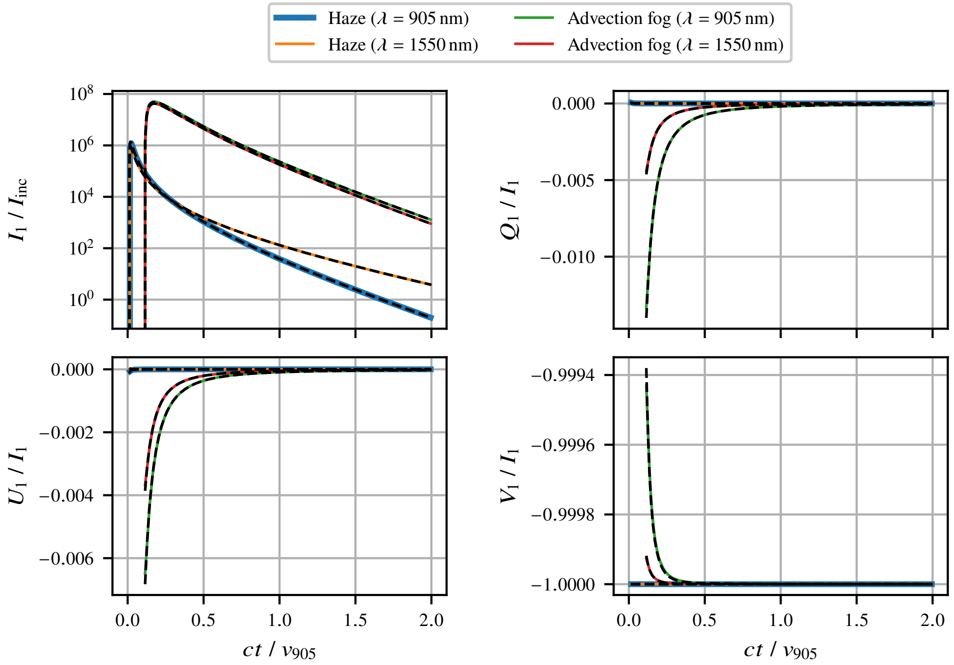

Figure S1. Stokes parameters I_1, Q_1, U_1, V_1 of the single scattered radiance from a point-like cone source. The radiance is shown for haze and advection fog at wavelengths of 905 nm and 1550 nm. Curves that would be hidden by other curves are drawn thicker. Analytic vRTE solutions are overlaid as dashed lines.

Table S2. Time-integrated Stokes parameter $\int_0^\infty I_0(t)\,\mathrm{d}t$ of the ballistically returned radiance from a point-like cone source with a perpendicularly illuminated Lambertian disc target in the beam path. The radiance is given for haze and advection fog at wavelengths of 905 nm and 1550 nm. I_0 exhibits a narrow peak at a time that corresponds to twice the target distance, whereas Q_0, U_0, and V_0 are zero due to the depolarizing target surface.

Fog type	$\int_0^\infty I_0(t)\,\mathrm{d}t \,/\, (I_{\mathrm{inc}} \cdot 1\,\mathrm{m}/c)$
Haze (λ = 905 nm)	7.956×10^5
Haze (λ = 1550 nm)	4.482×10^6
Advection fog (λ = 905 nm)	6.896×10^7
Advection fog (λ = 1550 nm)	6.232×10^7

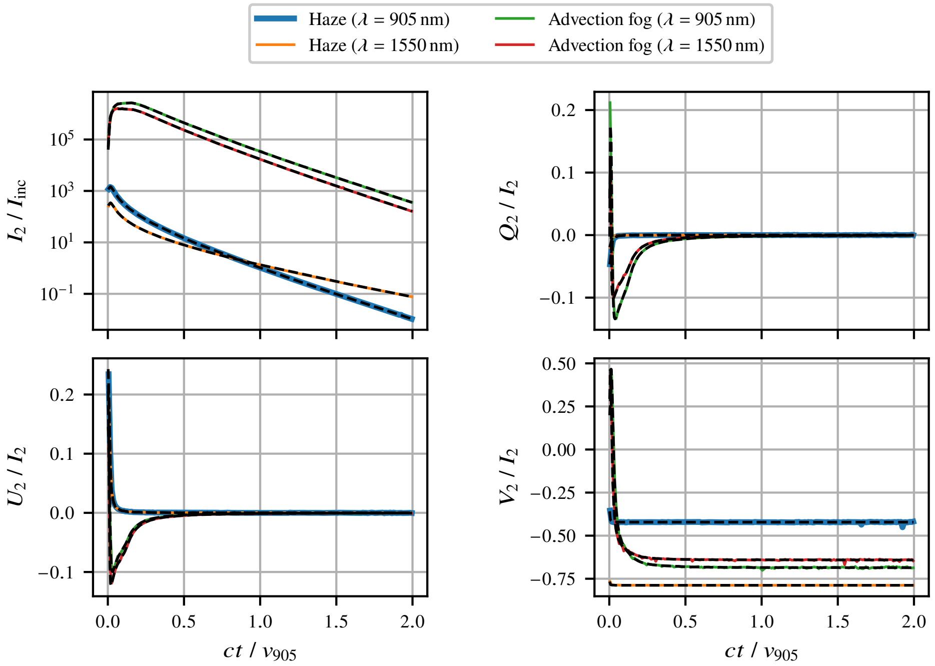

Figure S2. Stokes parameters I_2, Q_2, U_2, V_2 of the double scattered radiance from a point-like cone source. The radiance is shown for haze and advection fog at wavelengths of 905 nm and 1550 nm. Analytic vRTE solutions are overlaid as dashed lines.

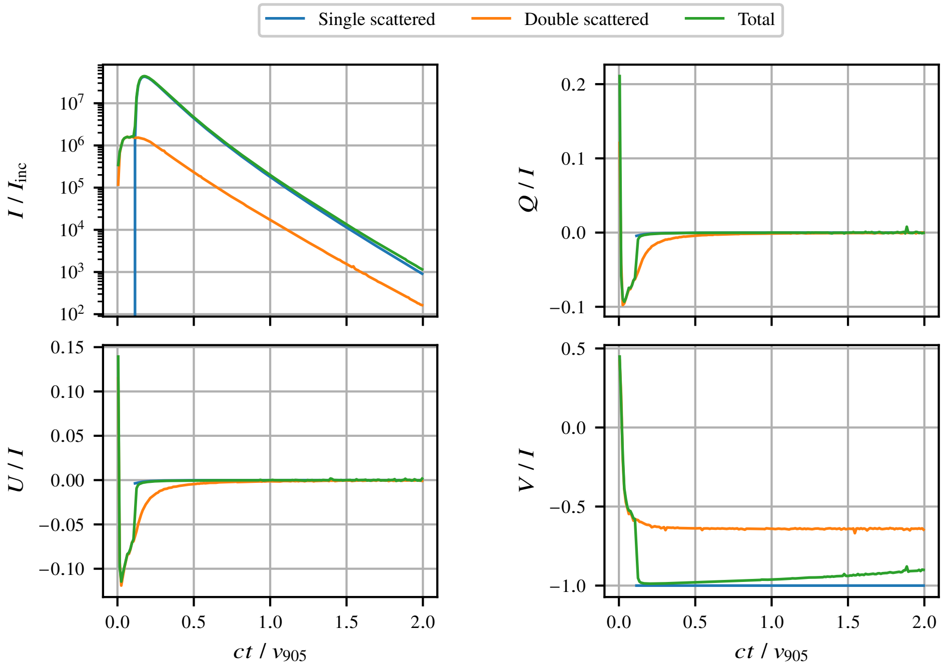

Figure S3. Stokes parameters of the single scattered radiance, the double scattered radiance, and the total radiance from a point-like cone source. The radiance is shown for advection fog at a wavelength of 1550 nm.

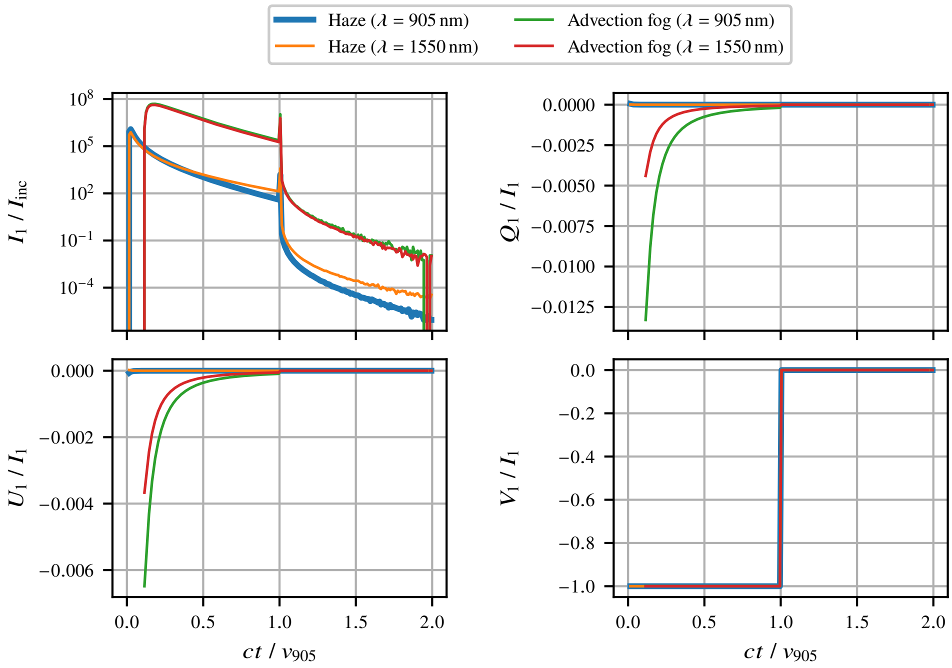

Figure S4. Like Figure S1, with a perpendicularly illuminated Lambertian disc target in the beam path. For V_1, all curves coincide.

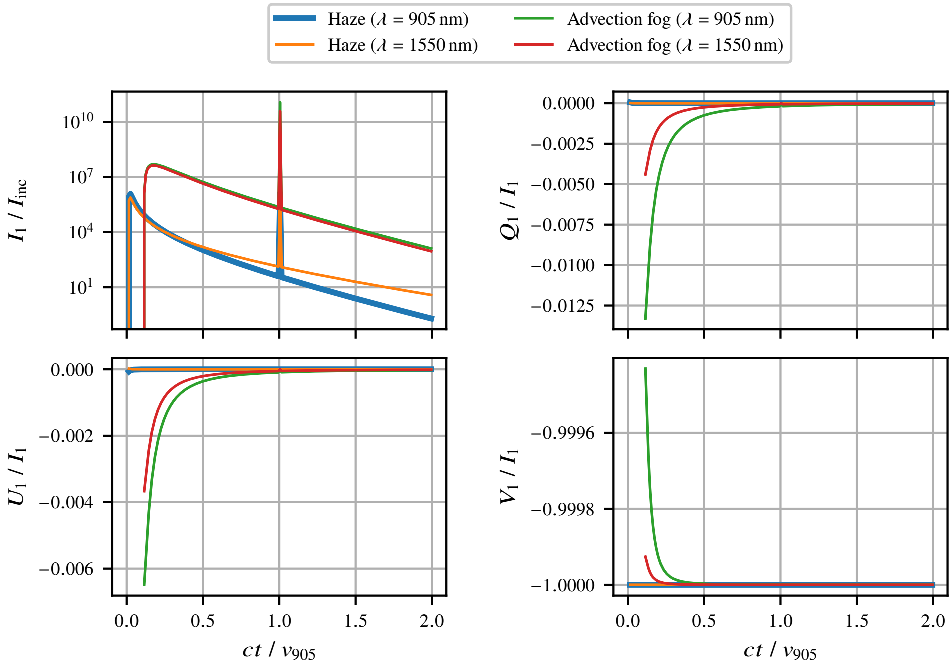

Figure S5. Like Figure S1, with a perpendicularly illuminated reflecting disc target in the beam path.

Table S3. Like Table S2, with a tilted (45°) Lambertian disc target in the beam path.

Fog type	$\int_0^\infty I_0(t)\,\mathrm{d}t\ /\ (I_{\mathrm{inc}} \cdot 1\,\mathrm{m}/c)$
Haze (λ = 905 nm)	4.839×10^5
Haze (λ = 1550 nm)	2.726×10^6
Advection fog (λ = 905 nm)	4.877×10^7
Advection fog (λ = 1550 nm)	4.406×10^7

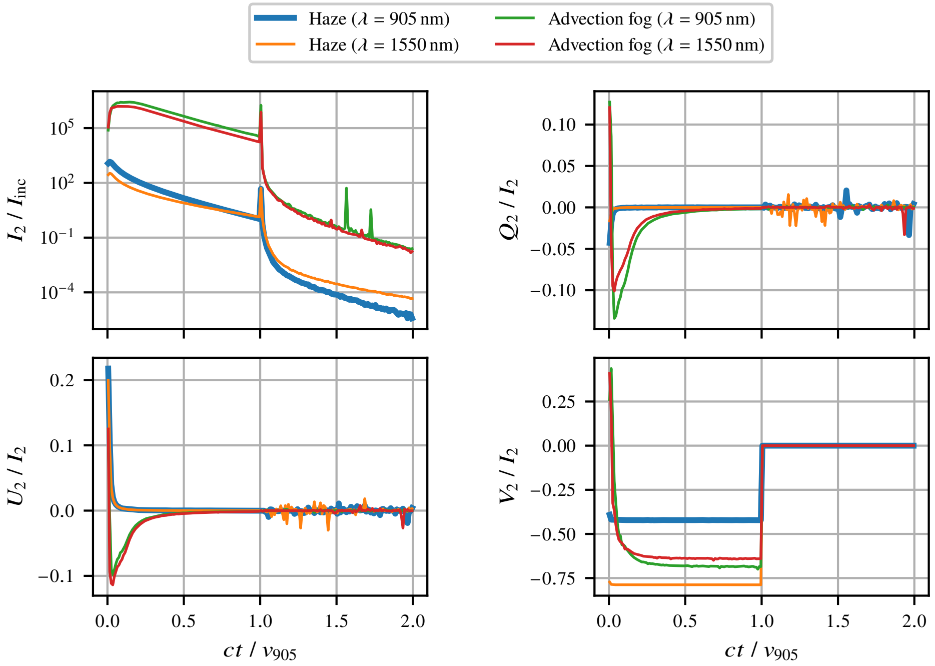

Figure S6. Like Figure S2, with a perpendicularly illuminated Lambertian disc target in the beam path.

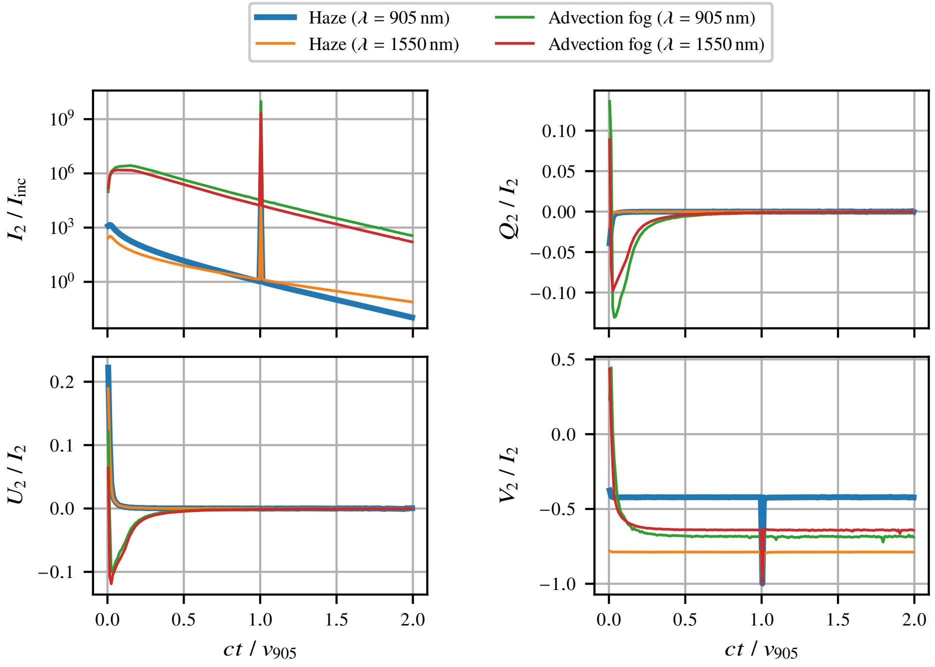

Figure S7. Like Figure S2, with a perpendicularly illuminated reflecting disc target in the beam path.

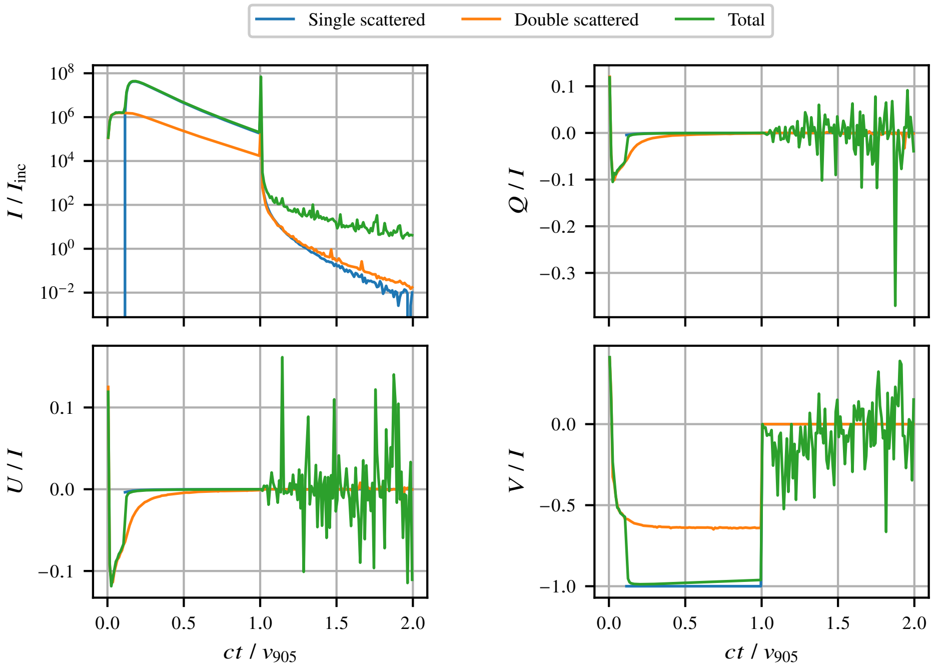

Figure S8. Like Figure S3, with a perpendicularly illuminated Lambertian disc target in the beam path.

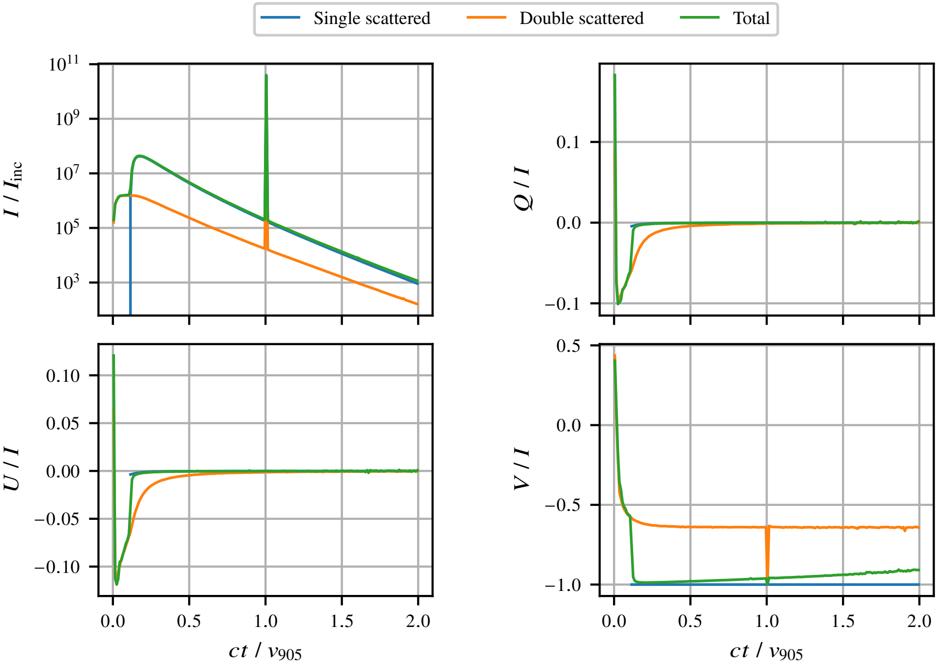

Figure S9. Like Figure S3, with a perpendicularly illuminated reflecting disc target in the beam path.

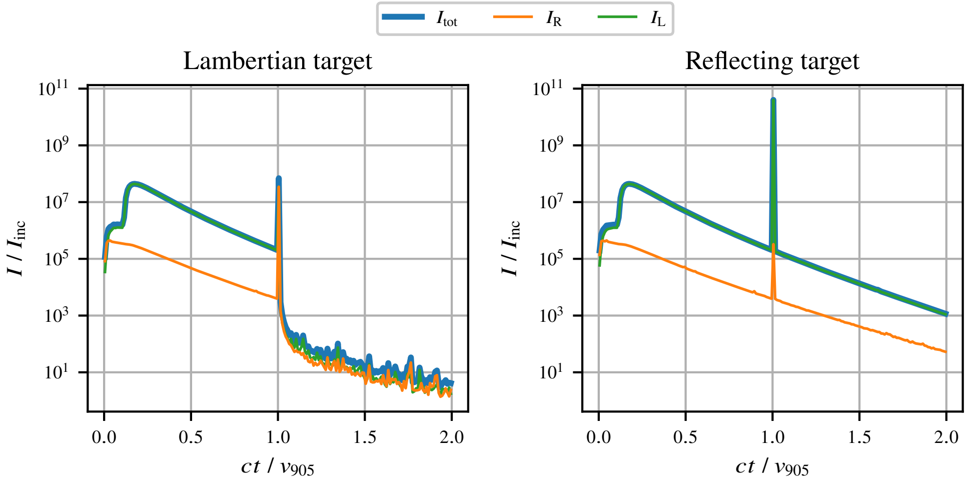

Figure S10. Total (I_{tot}), right-circularly polarized (I_R), and left-circularly polarized radiance (I_L) from a right-circularly polarized cone source with perpendicularly illuminated Lambertian (left) and reflecting (right) targets. The radiance is shown for advection fog at a wavelength of 1550 nm.

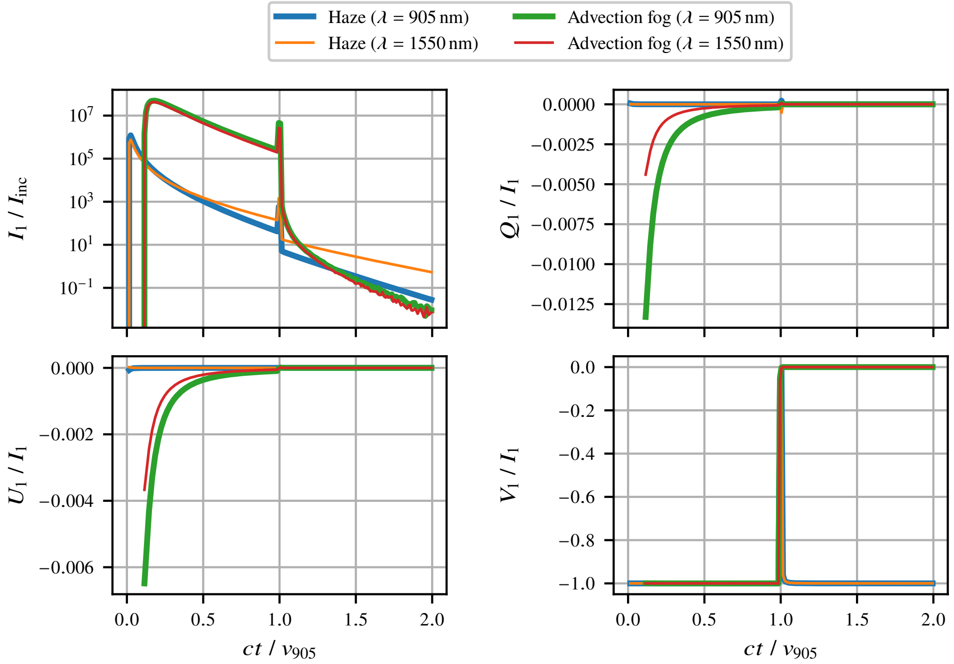

Figure S11. Like Figure S1, with a tilted Lambertian disc target in the beam path.

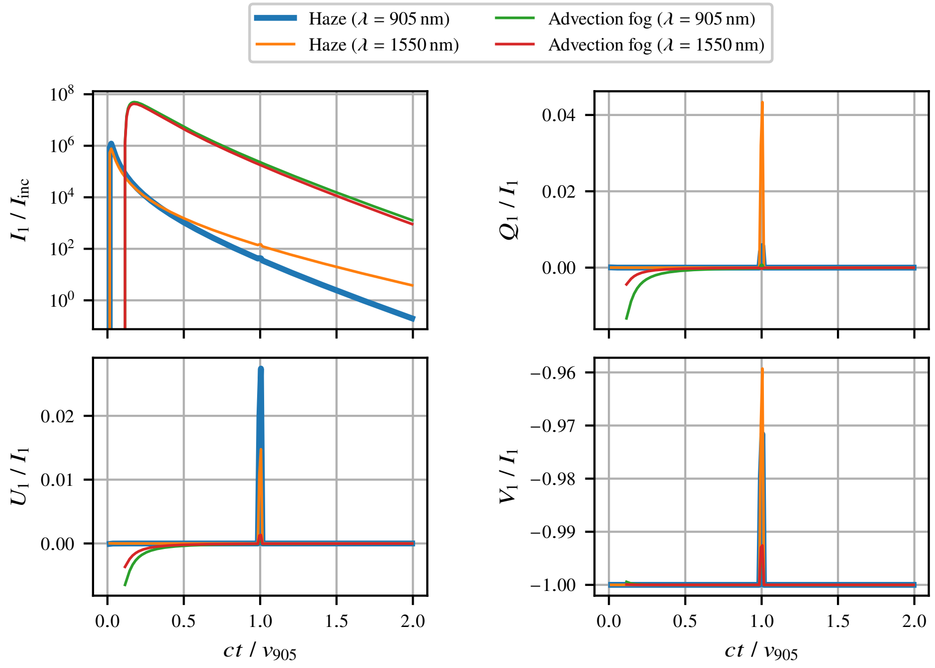

Figure S12. Like Figure S1, with a tilted reflecting disc target in the beam path.

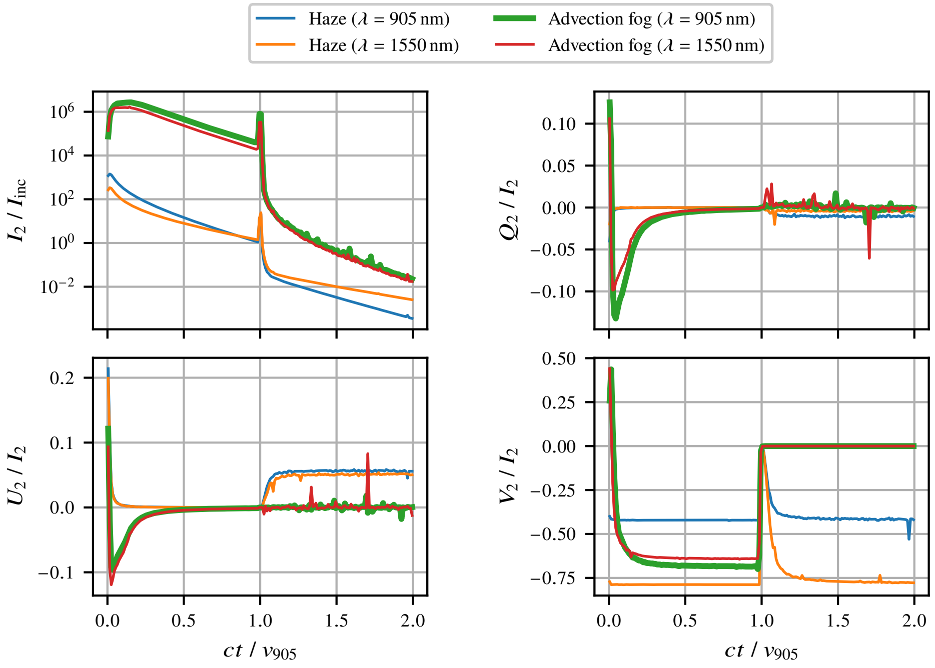

Figure S13. Like Figure S2, with a tilted Lambertian disc target in the beam path.

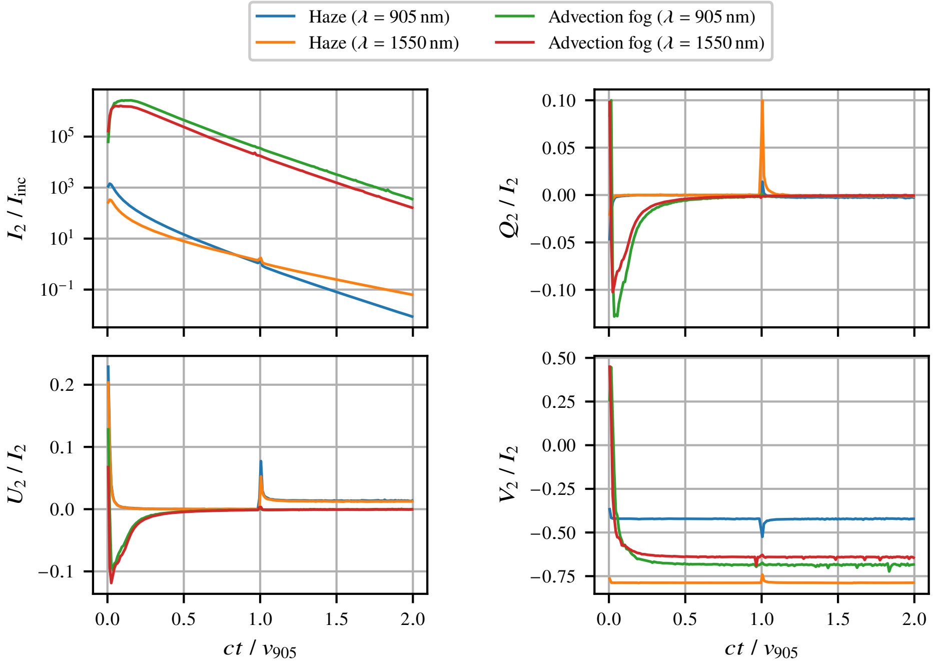

Figure S14. Like Figure S2, with a tilted reflecting disc target in the beam path.
